# Cross-national validation of the Depression, Anxiety and Stress Scale for Youth (DASS-Y) in Lebanese and Chinese adolescents

**DOI:** 10.1371/journal.pone.0360807

**Published:** 2026-10-21

**Authors:** Changhong Huang, Xiu-Mei Chen, Feten Fekih-Romdhane, Sahar Obeid, I-Hua Chen, Xiao-Ling Liao, Ping Xu, Zeng-Han Lee, Souheil Hallit, Olga Malas

**Affiliations:** 1 Faculty of Education, Qufu Normal University, Qufu, China; 2 Center of Teaching Quality Monitoring and Assessment, Jining University, Jining, China; 3 School of Information Engineering, Shandong Youth University of Political Science, Jinan, China; 4 The Tunisian Center of Early Intervention in Psychosis, Department of Psychiatry “Ibn Omrane”, Razi Hospital, Manouba, Tunisia; 5 Tunis El Manar University, Faculty of Medicine of Tunis, Tunis, Tunisia; 6 Department of Social and Education Sciences, School of Arts and Sciences, Lebanese American University, Jbeil, Lebanon; 7 Chinese Academy of Education Big Data, Faculty of Education, Qufu Normal University, Qufu, China; 8 Graduate School, Stamford International University, Bangkok, Thailand; 9 Faculty of Education, Jiangxi Science and Technology Normal University, Nanchang, China; 10 Sports Psychology Department, Guangzhou Sport University, Guangdong, China; 11 School of Education and Psychology, Minnan Normal University, Zhangzhou, Fujian, China; 12 Stamford International University, Bangkok, Thailand; 13 School of Medicine and Medical Sciences, Holy Spirit University of Kaslik, Jounieh, Lebanon; 14 Applied Science Research Center, Applied Science Private University, Amman, Jordan; 15 Department of Psychology, Sociology and Social Work, University of Lleida, Lleida, Spain; Amrita Vishwa Vidyapeetham - Amritapuri Campus, INDIA

## Abstract

**Background:**

The Depression, Anxiety and Stress Scale for Youth (DASS-Y) has been validated in several countries, but its measurement equivalence between Middle Eastern and East Asian adolescents remains untested, and prior validations primarily relied on single analytic approaches without examining complex factor structures or brief screening forms. This study evaluated the DASS-Y in Lebanese adolescents enduring sociopolitical strain and Chinese adolescents under comparatively stable conditions.

**Methods:**

The sample comprised 758 Lebanese (*M* age = 15.95, *SD* = 1.78; 63.2% female) and 1,062 Chinese adolescents (*M* age = 16.83, *SD* = 0.59; 47.5% female). Psychometric properties were examined using confirmatory factor analysis, exploratory structural equation modeling (ESEM), and bifactor variants of both. Differential item functioning was assessed using Rasch analysis. Reliability was indexed by composite reliability (CR), average variance extracted (AVE), and person separation reliability.

**Results:**

Lebanese adolescents showed a largely unidimensional pattern, whereas an ESEM solution better represented the Chinese data. Reliability was strong in both samples (subscale CR = 0.91 to 0.95, AVE = 0.58 to 0.71, person separation reliability = 0.79 to 0.85; total CR = 0.97 in both samples; total AVE = 0.63 for the Lebanese and 0.61 for the Chinese sample). Several items, mainly in the stress subscale, functioned non-equivalently across samples. The 12-item short form performed comparably (subscale CR = 0.88 to 0.92, AVE = 0.65 to 0.73; total CR = 0.96 and 0.94; total AVE = 0.64 and 0.57, respectively).

**Conclusions:**

Lebanese adolescents’ responses reflected a unitary distress construct, whereas Chinese adolescents’ responses showed greater differentiation among depression, anxiety, and stress, though a general distress factor remained dominant in both samples. Reliability was high throughout, so this divergence is structural rather than a matter of measurement precision. The 12-item form offers a practical screening option pending further cross-national confirmation.

## 1. Introduction

Depression and anxiety are among the most common mental health problems in adolescence worldwide [1]. A meta-analysis of 96 studies from 29 countries estimated the prevalence of clinically relevant depressive symptoms at 21.3%, with a significant increase from 1989 to 2022 [2]. National trend data show the same trajectory: in the United States, anxiety rose from 7.1% to 9.2% and depression from 3.1% to 4.0% between 2016 and 2020 [3]. These trends are particularly concerning because childhood and adolescence are critical developmental periods in which approximately half of all lifetime mental disorders first emerge [4], and many persist into adulthood [5]. Adolescents also commonly report elevated stress, understood as persistent tension, irritability, and difficulty relaxing rather than as a diagnosable disorder [6]. Because stress is not a diagnostic category, it is rarely captured in prevalence estimates of the kind described above. Yet academic pressure, one of the most commonly reported chronic stressors in this age group, was associated with depression, anxiety, or mixed internalizing symptoms in 48 of the 52 studies covered by a recent systematic review, with samples drawn from Asia, Europe, North America, and Australia [1].

Accurate and valid assessment instruments are therefore essential for identifying young people who need help and for designing effective interventions. Although several well-established instruments have been developed specifically for youth populations, most were constructed around a single construct. Widely used examples include the Children’s Depression Inventory (CDI) [7] for depressive symptoms and the Screen for Child Anxiety Related Emotional Disorders (SCARED) [8] for anxiety symptoms. Such measures capture their target domain well, but they leave two unsatisfactory options: administering several instruments together, which increases respondent burden in school settings where assessment time is limited, or assessing one domain in isolation, which risks overlooking the substantial co-occurrence among internalizing symptoms at this age [9]. The Depression, Anxiety and Stress Scale for Youth (DASS-Y) [10] was developed to address this gap, assessing all three negative emotional states within a single 21-item instrument. Its treatment of stress as a dimension alongside depression and anxiety is therefore of particular relevance for adolescents facing chronic academic and interpersonal demands.

Initial validation studies of the DASS-Y have yielded promising psychometric properties across diverse national settings, including Australia [10], China [11–13], Indonesia [14,15], Iran [16], Lebanon [17], Poland [18], and Serbia [19]. However, two fundamental questions remain. First, given the high subscale intercorrelations reported in these studies (e.g., 0.97 between anxiety and stress in [13]), do the three constructs hold together as distinct dimensions, or do they collapse into a more general distress factor? Second, does the scale function equivalently across national groups, such that meaningful cross-national comparisons can be drawn? Answering them also raises a further one: whether a brief form of the instrument can serve more than one national setting. These have practical consequences. If the subscales do not separate, reporting distinct depression, anxiety and stress profiles claims more than the instrument can support, and the total score becomes the defensible unit of interpretation. If item parameters do not transfer, prevalence differences between countries cannot be distinguished from differences in how the items are understood, and single-country findings cannot be read within a common frame. Resolving both would place cross-national comparison on firmer footing, while a validated brief form would make repeated school screening feasible. The present study addresses these issues through a psychometric evaluation of the DASS-Y in two samples of Lebanese and Chinese adolescents, examining item-level functioning, competing factor structures, and cross-national equivalence within a single design. Lebanon and China were chosen because they share a collectivist cultural classification yet differ markedly in adolescents’ sociopolitical circumstances, which makes them a demanding test of whether shared cultural features are sufficient for measurement equivalence (Section 1.2).

### 1.1 The depression, anxiety and stress scale for youth (DASS-Y)

The development of a youth-specific version of the DASS was motivated by persistent limitations of the adult scales when applied to younger respondents. The original DASS and the DASS-21 [6] have served as standard instruments for measuring distress across age groups for decades. It is now the case, however, that the application of these adult instruments to adolescent samples has produced inconsistent psychometric findings. Some investigative teams have supported the oblique three-factor structure [20, 21]. Others, working with adolescent samples in Malaysia, Vietnam, and North Macedonia, have failed to differentiate depression, anxiety, and stress, and have instead proposed one- or two-factor solutions [22,23]. Taken together, these findings indicate that there are developmentally specific features of distress in younger respondents that the adult scale does not adequately capture [24].

Szabo and Lovibond [10] addressed this issue through the development of the DASS-Y. The revised scale retains the three-factor structure of the DASS-21 while adapting item content for younger respondents. These adaptations operate at several levels, including vocabulary, sentence complexity, and the concreteness of the experiences described. One illustration concerns the framing of individual items: whereas the adult version includes inertia items that ask respondents to judge how readily they can begin an activity (e.g., “I found it difficult to work up the initiative to do things”), the DASS-Y employs more concrete and emotionally direct content (e.g., “I hated my life”). In their original validation, DASS-Y scores were positively correlated with worry and physiological hyperarousal, and negatively correlated with positive affect and life satisfaction [10]. These associations reflect what the scale is designed to measure: three related but distinguishable emotional syndromes, namely depression (anhedonia and hopelessness), anxiety (physiological arousal and anticipatory fear), and stress (tension and irritability).

However, an important consideration is that subscale intercorrelations on the DASS-Y tend to be high, and the degree of overlap varies considerably across settings. In one Chinese adolescent validation, correlations between the depression and anxiety subscales exceeded 0.60 [11]; in a second, the three factors correlated between 0.85 and 0.97, with anxiety and stress the least distinguishable pair [13]. Reported values are more moderate elsewhere, ranging from 0.69 to 0.78 in Polish adolescents [18] and from 0.52 to 0.66 in Iranian children and adolescents [16], though even these approach the range at which discriminant validity becomes difficult to defend. This pattern raises the question of construct validity: whether the three subscales reflect distinct constructs or a single underlying distress dimension expressed through different symptom presentations. The distinction is particularly difficult to draw in adolescent samples, given that the capacity for emotional differentiation is still developing during this period [25]. The variation across settings further suggests that the answer may not be the same everywhere.

Notwithstanding this concern, evidence of acceptable psychometric properties has continued to accumulate across the national contexts listed above. That foundation nonetheless leaves the dimensionality question open, since only one study [19] has systematically compared the three-factor solution with alternative factorial representations.

### 1.2 Measurement invariance and the Lebanese-Chinese comparison

Whether a factorial representation established in one population also holds in another is a question of measurement invariance, which is foundational for cross-national scale validation. The comparison of scores across populations is meaningful only if the instrument measures the same construct in the same way across those populations. In the absence of demonstrated invariance, observed mean differences are confounded by measurement non-equivalence [26], and the distinction between substantive differences in distress and instrument-related differences cannot be drawn.

Evidence on the cross-national equivalence of the DASS-Y is correspondingly thin. Published DASS-Y evaluations were identified by searching PubMed, Web of Science, Scopus and PsycINFO for “DASS-Y”, “DASS-Youth” and “Depression Anxiety Stress Scale for Youth” in June 2026, supplemented by forward and backward citation searching of the original validation study [10]; a full inventory, coded by sample, analytic approach and level of invariance tested, is provided in S1 Table. Only one investigation has tested measurement invariance across countries, comparing Australian and Serbian adolescents [19]. Both samples were drawn from Western educational traditions and from Indo-European languages, so the comparison holds constant much of what might plausibly threaten equivalence. The remaining validations are single-country, and the invariance evidence they provide is internal to the population studied: across sex [17,18], across age band, educational level and school type [15], and across time and within-country subgroups of school-aged youth [12]. Such evidence is necessary but not sufficient. Consistency within a population offers limited assurance about whether item parameters transfer to a population with a different language and sociopolitical context.

Work in the two settings examined here has established the instrument’s properties within each population: an Arabic translation has been validated in Lebanese adolescents, together with a shortened form [17], and Chinese validations have been conducted in school-aged samples [11–13]. These studies provide the within-country foundation on which any cross-national comparison depends. What has not yet been tested is whether the item parameters obtained in one of these populations hold in the other, and until that is established it remains unknown whether the DASS-Y functions equivalently across markedly different national contexts.

Both Lebanon and China are conventionally classified as collectivist [27], and on standard cultural dimensional accounts they would be expected to yield broadly similar patterns of emotional expression and reporting. The lived realities of adolescents in the two contexts, however, differ markedly. Lebanon has experienced sustained political instability and a severe economic crisis [28], with a substantial portion of social support provided through sectarian religious networks of schools, hospitals, and welfare institutions [29]. Data collection in Lebanon took place in November 2023, a period of heightened tensions with Israel, so acute sociopolitical strain was layered onto an already chronic context. Chinese adolescents, by contrast, contend predominantly with academic competition and parental expectations [30], within a politically stable environment in which prevailing cultural norms favor emotional restraint and hierarchical relationships in family and educational settings [31,32]. The pairing therefore offers a demanding test of whether a shared cultural classification is sufficient to produce measurement equivalence.

An important caveat warrants explicit acknowledgement. In the present design, the national context and acute stress exposure were confounded. If the factor structure of the DASS-Y differs between Lebanese adolescents (experiencing both chronic and acute sociopolitical strain) and Chinese adolescents (whose stressors are largely achievement-related), these two influences cannot be statistically separated. Therefore, the present comparison should be read as a description of how the DASS-Y functions across two real-world national contexts at a specific historical moment, rather than as a clean separation of national-context effects from acute stress effects. With this caveat in mind, the comparison nonetheless informs both cross-national assessment practice and the broader theoretical question of whether emotional constructs are universal or context bound.

### 1.3 Complementary analytic approach

A further limitation of the existing DASS-Y literature is methodological. Item response theory (IRT) has been applied: Cao et al. [11] used Rasch analysis alongside confirmatory factor analysis (CFA) in Chinese students, and Kurnianto et al. [15] have reported a Rasch evaluation of an Indonesian version. Alternative factorial representations have been examined once, by Jovanović [19], who compared CFA, exploratory structural equation modeling (ESEM) and bifactor-ESEM solutions. The two have not been combined: studies applying IRT have done so within a single assumed factor structure, while the one study comparing competing structures did not model items individually. The relationship between general distress and specific symptoms, and the behaviour of the individual items that carry it, have therefore not been characterised in the same dataset. Work on IRT and bifactor models has emphasised this as a limitation of approaches resting on classical test theory (CTT) alone [33,34]. The present study addresses this by combining the two traditions in a single dataset, using Rasch-based IRT to characterise the functioning of individual items and CTT-based factor-analytic modelling to compare competing structural representations (CFA, ESEM, bifactor CFA, and bifactor ESEM), in line with recommendations to pair IRT with bifactor approaches and to compare such models systematically when validating multidimensional psychological scales [35,36].

Each framework contributes distinct information to the analysis. The CTT provides indices of overall reliability and validity in a form that is readily comparable with the existing literature [37]. Rasch-based IRT, by contrast, places items and persons on a common logit metric, yields item-level location (difficulty) parameters and fit statistics while holding discrimination constant across items, and supports the detection of differential item functioning (DIF) across groups [36,38]. In cross-national research, DIF detection is essential rather than supplementary, since observed group differences cannot be confidently distinguished from measurement artifacts [36,39]. ESEM and bifactor models address a further limitation of traditional CFA, which assumes a simple structure with each item loading on a single factor. This assumption is questionable for emotional distress, where depression, anxiety, and stress share considerable conceptual territory. ESEM accommodates theoretically plausible cross-loadings, whereas bifactor models partition variance into a general factor and orthogonal specific factors. Together, these approaches afford a more nuanced representation of multidimensional constructs. The combined application of these methods also provides a stronger empirical basis for developing an abbreviated screening version. A 12-item Arabic short form already exists. Obeid et al. [17] derived the DASS-Y-12 by selecting the items corresponding to the adult DASS-12, but it was constructed within one national sample, by mapping onto an adult instrument rather than by empirical selection from the youth scale’s own item properties, and its behaviour outside Arabic-speaking adolescents is unknown.

### 1.4 Current study

The literature reviewed above leaves three matters unresolved. First, the high subscale intercorrelations reported across national settings leave open whether the DASS-Y captures three dimensions or a single distress construct. Second, its cross-national equivalence has been tested only once, between Australian and Serbian adolescents, so it remains unknown whether item parameters obtained in one national context hold in another. Neither question has yet been addressed with the combination of item-level and structural evidence that a firm answer requires. A third, practical matter follows: the one existing short form was derived within a single national sample and has not been evaluated elsewhere.

These gaps translate into four interrelated aims: (a) to evaluate item properties through Rasch analysis; (b) to compare competing factor structures (unidimensional, oblique CFA, ESEM, bifactor CFA, and bifactor ESEM) across the Lebanese and Chinese samples and to identify the optimal factorial representation of psychological distress in each context; (c) to develop and validate a shortened version suitable for efficient screening, and to compare it with the existing DASS-Y-12 [17]; and (d) to examine measurement equivalence across the two samples at both the item level (via DIF analysis) and the scale level (via multi-group CFA), as a prerequisite for valid cross-national mean comparisons.

## 2. Method

### 2.1 Participants

The Lebanese sample comprised 758 adolescents aged 12–18 years (*M* = 15.95, *SD* = 1.78), of whom 279 (36.8%) were male and 479 (63.2%) were female, all enrolled in middle or secondary school. The Chinese sample comprised 1,062 adolescents aged 15–18 years (*M* = 16.83, *SD* = 0.59), of whom 558 (52.5%) were male and 504 (47.5%) were female, all enrolled in senior secondary school. Mean age was comparable across the two samples, but the Lebanese sample was more heterogeneous in age and included younger adolescents, whereas the Chinese sample comprised a single grade cohort.

Both samples were non-clinical community samples of school-attending adolescents. No participants were recruited from clinical or psychiatric settings, and no diagnostic assessment or clinical interview was administered. The present study evaluates the measurement properties of the DASS-Y; its diagnostic accuracy could not be assessed, as no criterion measure was available against which sensitivity and specificity could be estimated. Additional sociodemographic variables, such as household income and parental education, were not collected in either protocol to limit participant burden and to keep the two protocols comparable. The sampling frames nonetheless indicate the socioeconomic range represented: the Chinese sample was drawn entirely from urban, public, academic-track senior secondary schools, and therefore does not include rural, private or vocational students, while online recruitment in Lebanon required device ownership and internet access. These constraints are considered further in the Limitations.

### 2.2 Procedure

Ethical approval for the study protocol was obtained from the Ethics Committee of the School of Pharmacy, Lebanese International University (IRB reference: 2023RC-033-LIUSOP) and the Institutional Review Board of the Jiangxi Psychological Consultant Association (IRB reference: JXSXL-2022-CL15). Additional information regarding the ethical, cultural, and scientific considerations specific to inclusivity in global research is included in the Supporting Information (S1 Checklist). This study employed a comparative cross-national design with parallel data collection in Lebanon and China. Identical procedural principles were applied in both contexts, although specific recruitment methods were adapted to the institutional and sociopolitical conditions of each country’s context.

Data collection in Lebanon was conducted from November 1–30, 2023, and in China from March 1–25, 2024. The inclusion criteria were identical across the two samples: residence in the respective country, proficiency in the language of the questionnaire (Arabic or Chinese), and age between 12 and 18 years. Although the eligible age range was identical, Chinese recruitment was directed at a single grade level (see below), so the Chinese sample did not span the full range. Participation was voluntary, and no compensation was provided. The decision not to offer compensation was consistent with the recommendations of both ethics committees, which aimed to reduce undue inducement and preserve the educational and clinical character of the survey. Parental or guardian consent was obtained prior to participation in both countries, in the form approved by the respective ethics committee (digital consent in Lebanon; documented oral consent in China, as described below), and the adolescents subsequently provided their own assent before accessing the questionnaire items. All items required a response to submit the survey; consequently, no missing data were present in either sample.

The sociopolitical and institutional conditions of the two countries necessitated different recruitment strategies for the study. In Lebanon, the regional security situation in November 2023, including intermittent school closures and movement restrictions, made centralized school-based recruitment impractical. Therefore, a snowball sampling approach conducted through social media was adopted, with the aim of reaching adolescents in all Lebanese governorates rather than a single locality. In China, by contrast, school attendance was uninterrupted, which permitted standardized administration in school computer laboratories with a sample of secondary school students drawn from two provinces.

Specifically, the Lebanese protocol employed online recruitment using a combination of convenience and snowball sampling methods. The questionnaire was administered through Google Forms and disseminated via social media platforms (WhatsApp, Instagram, and Facebook). Members of the research team distributed the survey link through their personal networks across different Lebanese governorates, asking acquaintances to forward the link to eligible adolescents. The survey landing page provided information on the study’s purpose, the voluntary nature of participation, confidentiality protections, and participant rights. Parents or legal guardians were required to read the study information and provide digital consent before the adolescent could proceed, after which the adolescents provided their own assent prior to accessing the questionnaire items. Participants were invited to share the link with peers who met the inclusion criteria, allowing recruitment to propagate through adolescent social networks. Because recruitment was not school-based, no sampling frame was available and a participation rate cannot be computed for the Lebanese sample.

The Chinese protocol employed a school-based recruitment strategy. The research team approached head teachers at secondary schools in the provincial capitals of one coastal and one central inland province, and five secondary schools agreed to participate in the study: three in Guangzhou, Guangdong Province, and two in Nanchang, Jiangxi Province. All five were public schools situated in urban districts, and all offered the academic (rather than vocational) senior secondary track. Approximately 3,000 grade 11 students were enrolled across the five schools. Within each school, intact classes at grade 11 were selected by school administrators, and all students in the selected classes were invited to participate. School teachers introduced the study to the students and informed them of their rights, including the voluntary nature of participation and the anonymous handling of data. Teachers obtained active oral consent from parents prior to administration and kept a record of it; written parental consent was not feasible within the participating schools’ administrative procedures, and the oral procedure was approved by the Institutional Review Board of the Jiangxi Psychological Consultant Association. Students subsequently completed the survey, administered online via Wenjuanxing, in their school computer laboratories during their lunch breaks. Study information was displayed on the survey landing page, and participants were required to provide assent before accessing the questionnaire items.

### 2.3 Instruments

The DASS-Y comprises 21 items distributed equally across three subscales (seven items each for depression, anxiety, and stress) and assesses the severity of symptoms over the preceding week. Each item is rated on a four-point Likert scale ranging from 0 (not true of me) to 3 (very true of me), with intermediate options of 1 (a little bit true of me) and 2 (fairly true of me). The subscale scores range from 0 to 21, with higher scores reflecting more severe symptoms. The clinical cut-off scores for the DASS-Y are 6 for depression, 5 for anxiety, and 11 for stress [10]. A total score above 23 has been recommended as an indicator of clinically significant psychological distress [10].

The previously published and validated Arabic [17] and Chinese [11] versions of the DASS-Y were administered, and no new translation was undertaken for the present study. In each case, the English original was translated by independent bilingual translators, and back-translation was performed by a further translator who had not participated in the forward stage and who worked without access to the original English version. An expert committee then compared the original and back-translated versions and resolved discrepancies: for the Arabic version, two psychiatrists, a psychologist and the research team; for the Chinese version, the translators, a Chinese language teacher, and specialists in pediatrics, public health and psychometrics. Both versions were pilot-tested with adolescents to confirm comprehension before use. The psychometric properties of both versions in the present samples are reported in the Results section.

### 2.4 Data analysis

Statistical analyses were conducted using Jamovi version 2.7.29. Descriptive statistics for the DASS-Y items were computed first, together with prevalence estimates of clinically significant symptoms based on the recommended cutoff scores. Spearman correlation coefficients were calculated to evaluate bivariate associations among items.

Rasch analysis was conducted to evaluate the psychometric properties of the DASS-Y items. Consistent with the Rasch model, item discrimination was constrained to be equal across items, so that only item location (difficulty) and person ability were estimated. Infit (information-weighted) and outfit (outlier-sensitive) mean square values between 0.50 and 1.50 were considered acceptable. Values below 0.50 indicate overfitting, reflecting item redundancy or local dependence, whereas values above 1.50 indicate underfitting, reflecting inconsistent or noisy response patterns.

Factor analytic modelling was then performed to evaluate the structure of the instrument. Given that all 21 DASS-Y items showed significant deviation from normality (Shapiro–Wilk tests, all *p*s < .001), maximum likelihood estimation with robust standard errors (MLR) was employed. MLR is appropriate for non-normal data and yields accurate parameter estimates with ordinal variables. Several competing factor structures were specified and compared: (a) a unidimensional model representing general psychological distress; (b) a correlated three-factor model in which depression, anxiety, and stress were modelled as distinct but correlated dimensions; (c) an ESEM permitting cross-loadings among factors; (d) a bifactor model with one general factor and three specific factors; and (e) a bifactor-ESEM model combining bifactor structure with cross-loadings. This comparative approach was adopted to identify the factorial representation that best characterized psychological distress in each national context. Model fit was evaluated against conventional thresholds: Comparative Fit Index (CFI) and Tucker-Lewis Index (TLI) values ≥0.90 indicating acceptable fit and ≥0.95 indicating excellent fit; Root Mean Square Error of Approximation (RMSEA) values ≤0.08 indicating acceptable fit and ≤0.05 indicating excellent fit; and Standardized Root Mean Square Residual (SRMR) values ≤0.08 indicating good fit [40]. Reliability was assessed using composite reliability (CR), which is equivalent to McDonald’s ω for congeneric item sets, together with average variance extracted (AVE). Both were computed from the standardized loadings of the oblique three-factor model for the subscales and the one-factor model for the total scale. These two models were used because CR and AVE are defined for simple structure, in which each item loads on a single factor; under ESEM and bifactor specifications, item variance is distributed across cross-loadings or partitioned between general and specific factors, so subscale coefficients are no longer interpretable in the conventional way. Estimating them from the simple-structure models also matches how DASS-Y scores are computed in practice, since users sum the items of each subscale and of the scale as a whole regardless of which model best represents the data. Values of CR > 0.70 and AVE > 0.50 were taken as satisfactory [41].

In addition to the full 21-item DASS-Y, an abbreviated 12-item version was also developed. The same set of psychometric analyses was conducted on this abbreviated instrument to evaluate its factor structure and reliability.

Measurement invariance between the Lebanese and Chinese samples was examined. DIF was assessed within the Rasch framework using item difficulty contrasts, with contrasts ≥ 0.64 logits (approximately 1.5 ETS delta units) [42] classified as moderate to large, following the Educational Testing Service criteria. In addition to the DIF analysis, a multi-group CFA was conducted to test measurement invariance at the scale level, following the standard configural, metric, and scalar invariance sequence. Changes in model fit indices (ΔCFI > −0.010, ΔRMSEA ≤ 0.015, and ΔSRMR ≤ 0.030 for loadings and ≤ 0.010 for intercepts) were evaluated against established criteria [43,44] to determine whether imposing equality constraints on factor loadings and item intercepts across the two samples significantly worsened the model fit. Latent mean comparisons across the two samples were conducted only after the measurement invariance was formally established.

Finally, two supplementary sets of invariance analyses were conducted within samples. Because socioeconomic status was not measured directly, the first tested invariance across the two Chinese provincial subsamples, which differ substantially in level of economic development. The second, prompted by the uneven sex distribution across the Lebanese and Chinese samples, tested invariance across male and female adolescents in each country.

## 3. Results

### 3.1 Descriptive statistics and inter-item correlations

Tables 1 and 2 report the means and standard deviations for each DASS-Y item for Lebanese and Chinese participants separately. Based on the established DASS-Y cutoff scores, 52.2% of the Lebanese participants met the criteria for mild or above depression (*n* = 396), 55.9% met the criteria for anxiety (*n* = 424), and 26.5% met the criteria for stress (*n* = 201). For the Chinese participants, the corresponding proportions were lower: 27.0% (*n* = 287), 24.9% (*n* = 265), and 6.8% (*n* = 72). Taking the scale as a whole, 46.0% of Lebanese adolescents (*n* = 349) exceeded the DASS-Y total score cutoff of 23, whereas only 12.7% (*n* = 135) of Chinese adolescents did so.

**Table 1:** Descriptive Statistics and Spearman Correlations of the DASS-Y for Lebanese Participants (*N* = 758).

	*Mean*	*SD*	1	2	3	4	5	6	7	8	9	10	11	12	13	14	15	16	17	18	19	20	21
1. Item 3	1.05	0.97	—																				
2. Item 5	0.90	0.98	0.69	—																			
3. Item 10	0.97	0.97	0.65	0.66	—																		
4. Item 13	1.01	0.97	0.66	0.68	0.71	—																	
5. Item 16	0.89	0.95	0.64	0.72	0.71	0.69	—																
6. Item 17	0.95	0.93	0.66	0.72	0.70	0.77	0.75	—															
7. Item 21	0.94	0.94	0.65	0.72	0.73	0.77	0.77	0.79	—														
8. Item 2	0.94	0.92	0.62	0.61	0.61	0.60	0.63	0.62	0.62	—													
9. Item 4	0.91	0.95	0.62	0.62	0.65	0.64	0.64	0.64	0.69	0.69	—												
10. Item 7	0.95	0.96	0.65	0.70	0.64	0.69	0.66	0.67	0.69	0.68	0.69	—											
11. Item 9	0.92	0.94	0.63	0.70	0.66	0.70	0.68	0.70	0.71	0.61	0.67	0.71	—										
12. Item 15	0.93	0.95	0.64	0.67	0.68	0.73	0.74	0.76	0.76	0.66	0.69	0.68	0.74	—									
13. Item 19	0.93	0.96	0.64	0.69	0.66	0.71	0.70	0.70	0.75	0.62	0.69	0.73	0.66	0.72	—								
14. Item 20	0.91	0.94	0.64	0.65	0.62	0.70	0.71	0.71	0.73	0.62	0.70	0.68	0.70	0.74	0.71	—							
15. Item 1	1.20	0.99	0.51	0.44	0.46	0.49	0.41	0.49	0.51	0.43	0.40	0.45	0.40	0.42	0.47	0.38	—						
16. Item 6	1.10	0.96	0.58	0.55	0.62	0.61	0.63	0.67	0.65	0.61	0.63	0.60	0.60	0.61	0.61	0.58	0.54	—					
17. Item 8	1.15	0.97	0.60	0.57	0.63	0.63	0.59	0.65	0.63	0.56	0.57	0.60	0.57	0.60	0.62	0.56	0.56	0.66	—				
18. Item 11	1.06	0.96	0.64	0.67	0.64	0.72	0.67	0.69	0.70	0.56	0.61	0.65	0.64	0.68	0.70	0.66	0.51	0.67	0.65	—			
19. Item 12	1.05	0.94	0.65	0.63	0.68	0.72	0.68	0.69	0.70	0.60	0.62	0.66	0.62	0.67	0.68	0.66	0.53	0.68	0.69	0.73	—		
20. Item 14	0.94	0.94	0.60	0.64	0.66	0.66	0.68	0.68	0.70	0.60	0.63	0.65	0.64	0.69	0.67	0.66	0.47	0.66	0.62	0.62	0.67	—	
21. Item 18	1.05	0.92	0.61	0.61	0.64	0.71	0.64	0.72	0.70	0.60	0.61	0.63	0.63	0.70	0.67	0.65	0.55	0.68	0.65	0.71	0.72	0.65	—

Note. All correlation coefficients were significant at *p* < .001.

**Table 2:** Descriptive Statistics and Spearman Correlations of the DASS-Y for Chinese Participants (*N* = 1062).

	*Mean*	*SD*	1	2	3	4	5	6	7	8	9	10	11	12	13	14	15	16	17	18	19	20	21
1. Item 3	0.44	0.69	—																				
2. Item 5	0.55	0.75	0.67	—																			
3. Item 10	0.46	0.70	0.77	0.69	—																		
4. Item 13	0.45	0.69	0.74	0.67	0.71	—																	
5. Item 16	0.50	0.74	0.67	0.77	0.69	0.71	—																
6. Item 17	0.46	0.69	0.69	0.70	0.69	0.76	0.77	—															
7. Item 21	0.52	0.72	0.69	0.79	0.73	0.75	0.78	0.77	—														
8. Item 2	0.36	0.64	0.59	0.55	0.58	0.63	0.57	0.58	0.60	—													
9. Item 4	0.35	0.65	0.62	0.55	0.61	0.64	0.60	0.60	0.60	0.75	—												
10. Item 7	0.37	0.66	0.61	0.55	0.59	0.64	0.60	0.60	0.60	0.76	0.74	—											
11. Item 9	0.44	0.69	0.70	0.67	0.66	0.72	0.67	0.67	0.68	0.62	0.66	0.66	—										
12. Item 15	0.46	0.71	0.71	0.67	0.71	0.73	0.68	0.70	0.71	0.60	0.63	0.66	0.85	—									
13. Item 19	0.42	0.68	0.63	0.62	0.61	0.67	0.63	0.64	0.66	0.72	0.75	0.69	0.70	0.67	—								
14. Item 20	0.43	0.69	0.67	0.65	0.63	0.69	0.64	0.65	0.68	0.67	0.69	0.68	0.78	0.74	0.76	—							
15. Item 1	0.58	0.72	0.56	0.56	0.58	0.61	0.58	0.60	0.61	0.58	0.56	0.62	0.61	0.64	0.61	0.62	—						
16. Item 6	0.56	0.70	0.62	0.61	0.60	0.63	0.63	0.63	0.68	0.58	0.59	0.61	0.66	0.68	0.62	0.65	0.73	—					
17. Item 8	0.76	0.78	0.53	0.60	0.54	0.57	0.59	0.58	0.63	0.43	0.44	0.48	0.56	0.57	0.50	0.52	0.55	0.56	—				
18. Item 11	0.53	0.70	0.59	0.56	0.58	0.59	0.61	0.58	0.61	0.57	0.59	0.57	0.59	0.61	0.58	0.60	0.69	0.73	0.52	—			
19. Item 12	0.67	0.77	0.50	0.53	0.46	0.48	0.51	0.48	0.54	0.38	0.40	0.43	0.50	0.49	0.44	0.46	0.46	0.50	0.64	0.44	—		
20. Item 14	0.76	0.79	0.50	0.53	0.51	0.49	0.53	0.48	0.56	0.45	0.46	0.46	0.49	0.50	0.49	0.47	0.52	0.50	0.66	0.48	0.60	—	
21. Item 18	0.75	0.80	0.58	0.64	0.57	0.59	0.61	0.60	0.64	0.49	0.51	0.52	0.59	0.62	0.55	0.55	0.59	0.59	0.77	0.58	0.63	0.67	—

Note. All correlation coefficients were significant at *p* < .001.

With respect to inter-item relationships (see Tables 1 and 2), Spearman’s correlation analyses revealed strong associations among all DASS-Y items. The coefficients ranged from 0.40 to 0.79 for Lebanese participants and from 0.38 to 0.85 for Chinese participants. All correlations were statistically significant (*p* < 0.001).

### 3.2 Rasch analysis

The dimensionality was first examined at the whole-scale level. A principal component analysis (PCA) of the Rasch model residuals returned eigenvalues of 2.28 for the Lebanese sample and 3.01 for the Chinese sample. Both values exceeded the 1.50 threshold [45], indicating multidimensionality and supporting the decision to run the Rasch analysis separately for each subscale.

When PCA was conducted separately for each subscale, the first eigenvalues were all below 1.50, which confirmed the unidimensionality assumption needed for Rasch analysis [45]. The average absolute Q3 statistics ranged from 0.13 to 0.14 in the Lebanese sample and from 0.12 to 0.15 in the Chinese sample, all falling below the 0.20 threshold [46] and confirming the local independence assumption. Monotonicity of the scale was verified through person-item maps (see Figs 1–3), which showed that response category thresholds progressed in ordered sequence from left to right across all items. Higher trait levels thus corresponded systematically to endorsement of higher response categories.

**Fig 1:**
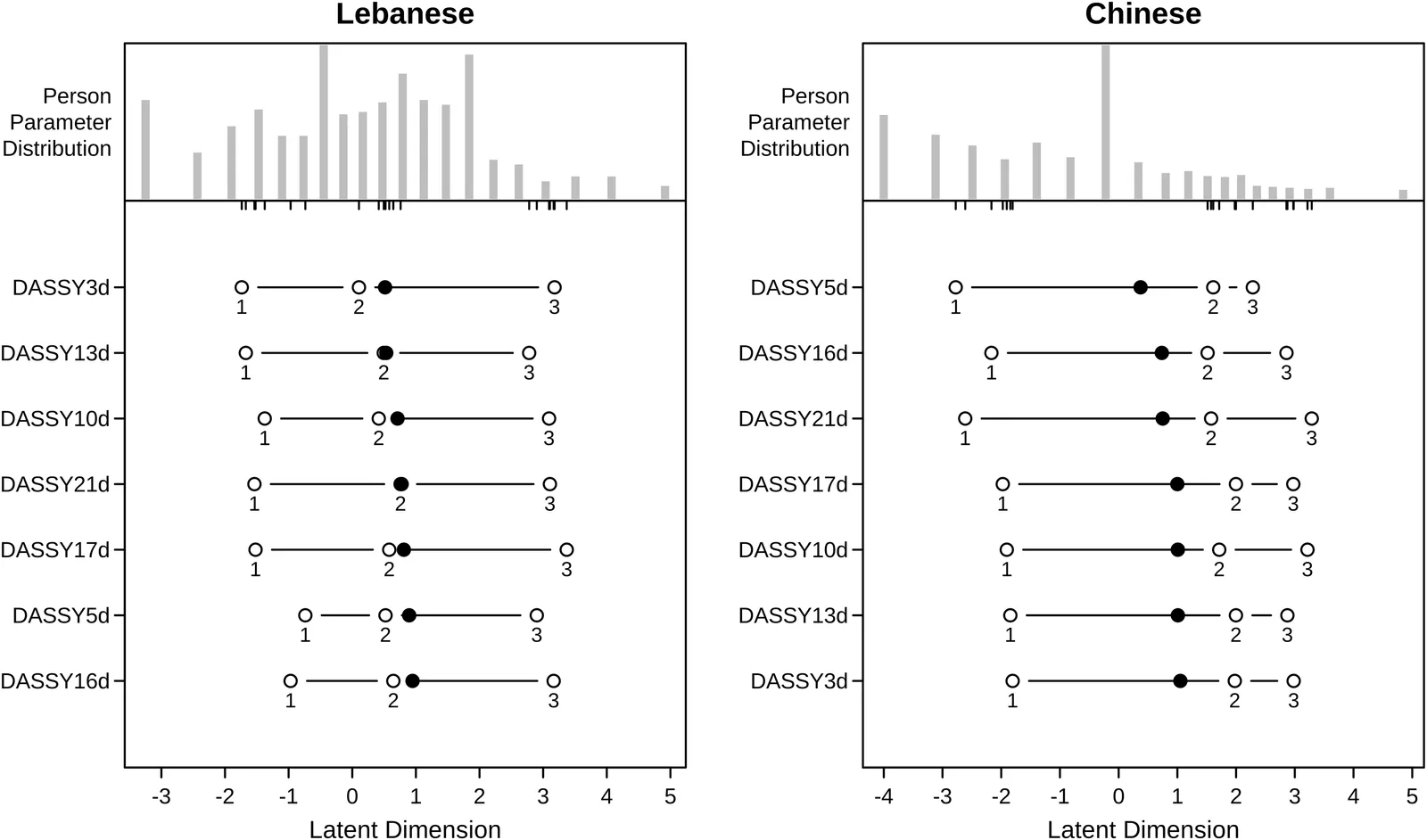
Person-Item Map for the Depression Subscale of the DASS-Y.

**Fig 2:**
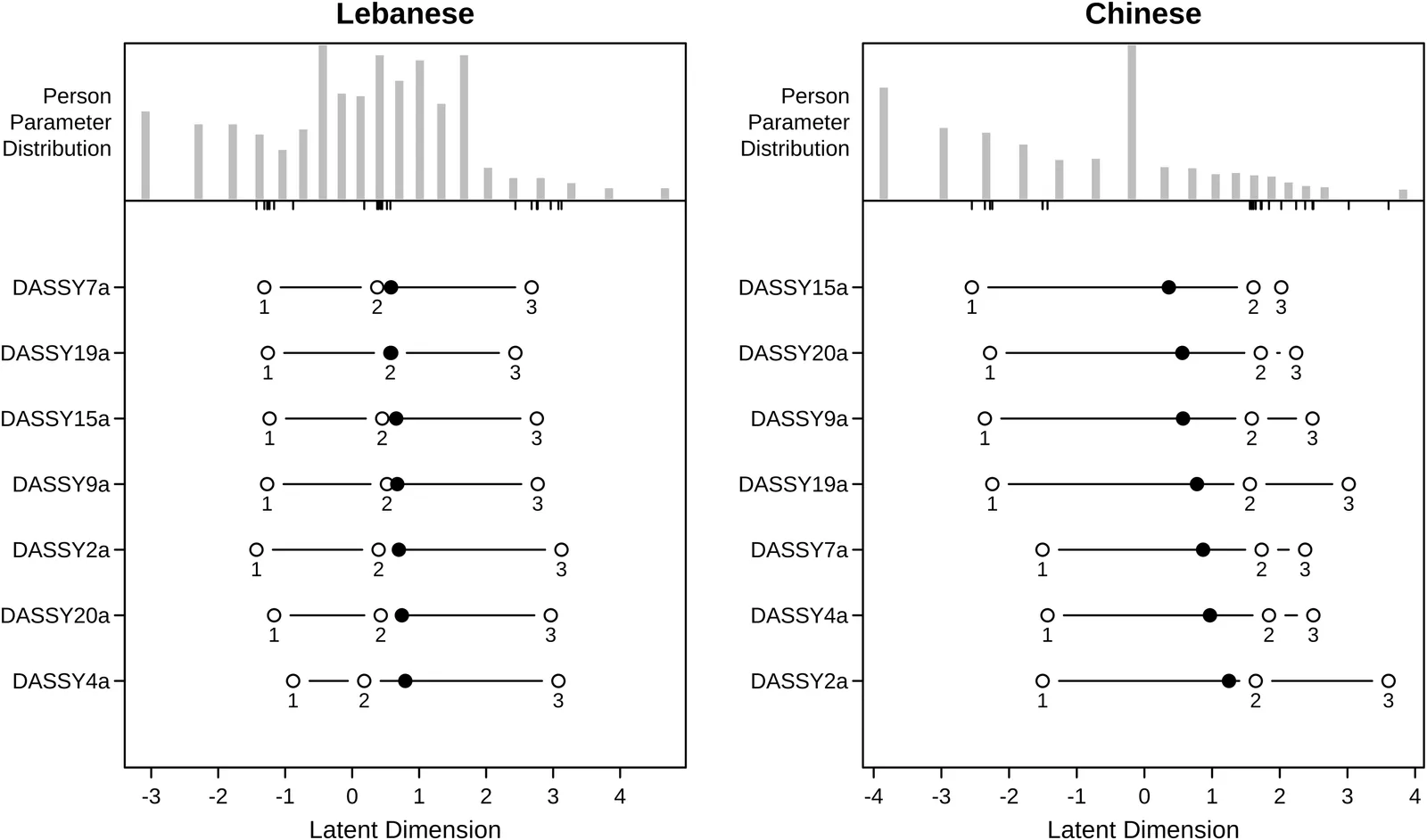
Person-Item Map for the Anxiety Subscale of the DASS-Y.

**Fig 3:**
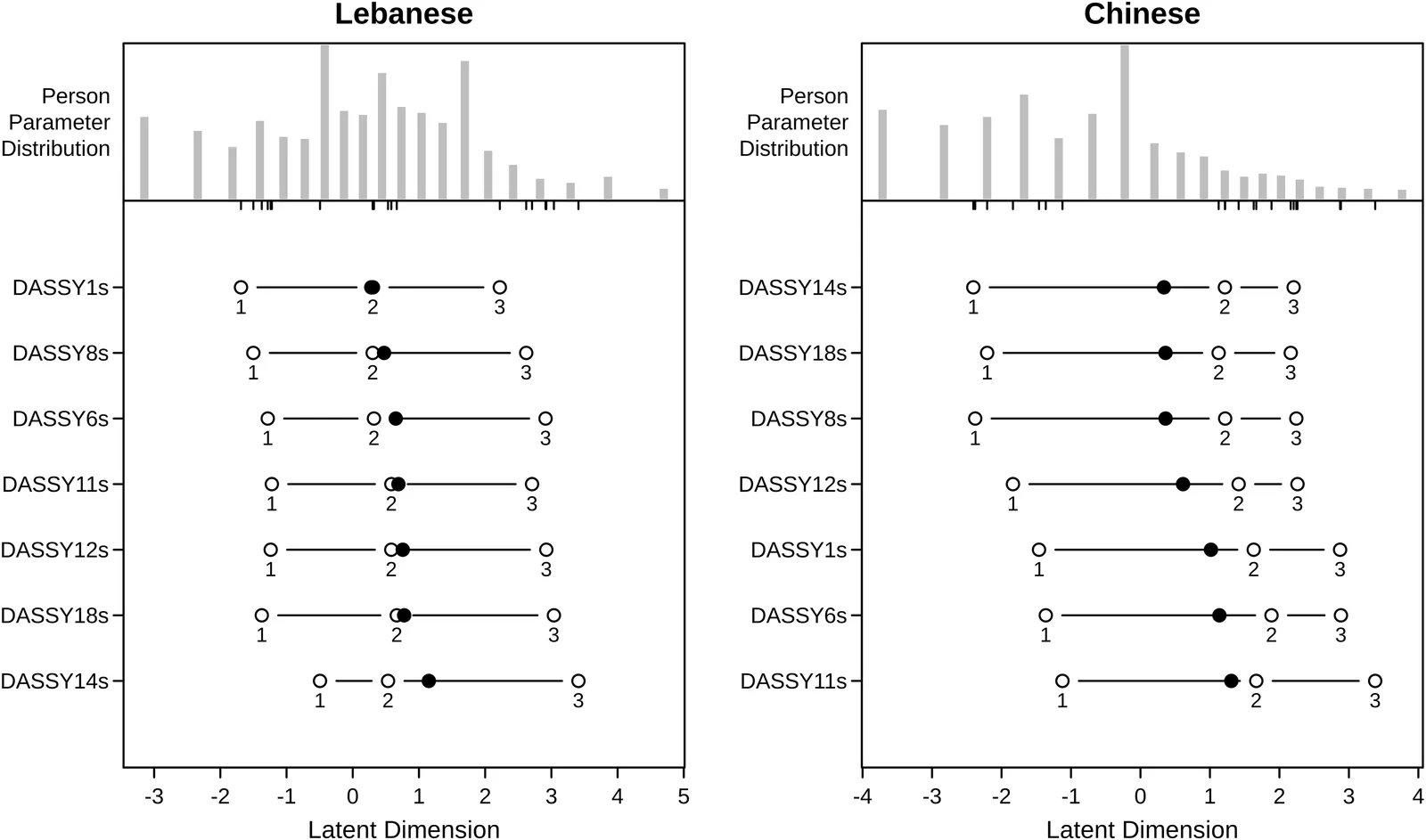
Person-Item Map for the Stress Subscale of the DASS-Y.

Table 3 reports the item psychometric properties, including infit/outfit mean square statistics and difficulty parameters. Nearly all DASS-Y items showed good item validity, with infit/outfit values falling within the acceptable 0.50 to 1.50 range. There was one exception: Item 1 of the stress subscale produced values outside this range for Lebanese participants. This indicated underfit and suggested inconsistent interpretation or noisy response patterns. Person separation reliability indices from the Rasch analysis showed adequate differentiation across all three subscales in both samples. The coefficients were 0.85, 0.81, and 0.83 for the Depression, Anxiety, and Stress subscales, respectively, in the Lebanese sample, and 0.82, 0.81, and 0.79 in the Chinese sample. Item separation reliability was 0.87, 0.92, and 0.93 for the Depression, Anxiety, and Stress subscales, respectively, in the Lebanese sample, and 0.93, 0.94, and 0.98 in the Chinese sample.

**Table 3:** Fit Statistics from Rasch Analysis for DASS-Y Items: Comparison Between Lebanese and Chinese Participants.

Source	Lebanese				Chinese				Absolute difficulty contrast
	Difficulty	Point-biserial	Infit MnSq	Outfit MnSq	Difficulty	Point-biserial	Infit MnSq	Outfit MnSq	
Subscale of Depression									0.41
Item 3. Did not enjoy anything	−0.27	0.82	1.38	1.41	0.32	0.84	1.12	1.07	0.59
**Item 5. Hated life**	0.16	0.82	1.01	1.03	−0.53	0.85	1.16	1.10	0.69
Item 10. Nothing nice to look forward to	−0.04	0.84	1.09	1.09	0.17	0.85	1.08	1.03	0.21
Item 13. Could not stop feeling sad	−0.22	0.86	0.99	0.99	0.28	0.85	0.97	0.93	0.50
Item 16. Hated myself	0.24	0.84	0.90	0.89	−0.13	0.86	1.05	0.98	0.37
Item 17. Felt like I was no good	0.08	0.87	0.83	0.85	0.21	0.86	0.90	0.85	0.13
Item 21. Felt life was terrible	0.05	0.87	0.81	0.79	−0.32	0.89	0.75	0.70	0.37
Subscale of Anxiety									0.28
Item 2. Felt dizzy, like about to faint	−0.04	0.80	1.19	1.26	0.46	0.81	1.18	1.19	0.50
Item 4. Trouble breathing/fast breathing	0.04	0.82	1.00	1.00	0.32	0.83	0.96	0.94	0.28
Item 7. Hands felt shaky	−0.09	0.84	0.95	0.95	0.13	0.82	1.04	1.06	0.22
Item 9. Felt terrified	0.03	0.83	0.99	1.02	−0.23	0.87	0.88	0.83	0.26
Item 15. Felt about to panic	0	0.84	0.89	0.86	−0.46	0.85	1.08	1.05	0.46
Item 19. Heart beating fast without exertion	0	0.83	1.02	0.99	0	0.87	0.91	0.89	0
Item 20. Scared for no good reason	0.05	0.83	0.96	0.95	−0.22	0.86	0.92	0.84	0.27
Subscale of Stress									0.56
**Item 1. Got upset about little things**	−0.46	0.73	1.54	1.68	0.34	0.77	1.02	1.00	0.80
Item 6. Over-reacted to situations	−0.04	0.83	0.90	0.89	0.50	0.77	0.94	0.90	0.54
Item 8. Stressing about lots of things	−0.24	0.82	0.97	0.95	−0.47	0.83	0.85	0.84	0.23
Item 11. Easily irritated	0.01	0.81	0.93	0.94	0.64	0.75	1.02	1.05	0.63
Item 12. Difficult to relax	0.09	0.84	0.79	0.77	−0.08	0.74	1.32	1.30	0.17
**Item 14. Got annoyed when interrupted**	0.53	0.78	1.02	0.97	−0.49	0.78	1.09	1.09	1.02
Item 18. Easily annoyed	0.11	0.83	0.84	0.81	−0.44	0.84	0.77	0.76	0.55

Note. Difficulty values are expressed in logits. MnSq = mean square. The difficulty contrast column reports the absolute difference in item difficulty between the Lebanese and Chinese samples; values in the shaded subscale rows are the mean absolute contrast for that subscale. Items shown in bold (Items 1, 5, and 14) exhibited contrasts exceeding 0.64 logits, indicating potential differential item functioning across the Lebanese and Chinese samples.

Analyses of difficulty patterns revealed notable cross-national differences, especially within the stress subscale. Three items exceeded the DIF criterion. The largest contrast by a substantial margin was Item 14 (“I got annoyed when people interrupted me”; 1.02 logits), followed by Item 1 (“I got upset about little things”; 0.80 logits) and Item 5 (“I hated my life”; 0.69 logits); Item 11 fell marginally below the criterion (0.63 logits) and was retained. The mean absolute contrast was 0.56 logits in the stress subscale, compared with 0.41 in depression and 0.28 in anxiety. Within the stress subscale, non-equivalence was concentrated in items describing irritability and over-reaction (Items 1, 6, 11, 14, 18; mean contrast 0.71 logits), whereas the two items describing tension and difficulty relaxing (Items 8 and 12) showed among the smallest contrasts in the instrument (mean 0.20). The direction of the largest differences was not uniform: Items 14 and 5 were markedly easier to endorse for Chinese adolescents relative to their overall trait level, whereas Item 1 was easier to endorse for Lebanese adolescents. These patterns suggest cross-national variation in the manifestation and interpretation of stress symptoms, reflecting a combination of national context, language-level differences in emotion vocabulary, and acute stress exposure rather than cultural differences alone.

### 3.3 Factor structure

Although Item 1 of the Stress subscale showed poor fit in the Rasch analysis, we retained it for the CFA to maintain comparability across samples. Table 4 reports the comparative fit indices for the factor structures in both countries. The one-factor structure achieved an acceptable model fit for the Lebanese participants but failed to achieve an adequate fit for the Chinese participants. In contrast, the oblique three-factor model showed acceptable fit indices in both national contexts. However, discriminant validity was a problem for this model. Interfactor correlations exceeded 0.90 in the Lebanese sample and ranged from 0.86 to 0.90 in the Chinese sample.

**Table 4:** Model Fit Indices for Competing Factor Structures of the 21-Item DASS-Y Across Lebanese and Chinese Participants.

	*χ*^*2*^ (*df*)	CFI	TLI	RMSEA; 90% CI	SRMR
**Lebanese**					
One-factor	481.35 (189)	0.957	0.952	0.045; 0.042-0.049	0.030
Three-factor	347.21 (186)	0.976	0.973	0.034; 0.030-0.038	0.026
Bifactor model	291.22 (168)	0.982	0.977	0.031; 0.027-0.035	0.021
ESEM	252.19 (150)	0.985	0.979	0.030; 0.025-0.035	0.017
Bifactor ESEM	193.68 (132)	0.991	0.985	0.025; 0.019-0.030	0.013
**Chinese**					
One-factor	1363.89 (189)	0.861	0.845	0.077; 0.074-0.079	0.052
Three-factor	856.12 (186)	0.921	0.910	0.058; 0.056-0.061	0.040
Bifactor model	575.11 (168)	0.952	0.940	0.048; 0.045-0.051	0.037
ESEM	590.99 (150)	0.948	0.927	0.053; 0.050-0.056	0.024
Bifactor ESEM	416.76 (132)	0.966	0.946	0.045; 0.042-0.048	0.020

CFI = comparative fit index; TLI = Tucker-Lewis Index; RMSEA = root mean square error of approximation; SRMR = standardized root mean square residual; CI = confidence interval

Turning to the more complex models, the ESEM yielded a substantial improvement in fit over the oblique three-factor model for the Chinese participants, with gains in CFI (ΔCFI = 0.027) and TLI (ΔTLI = 0.017), and reductions in SRMR (ΔSRMR = 0.016). Following Van Zyl and Ten Klooster’s [47] recommendations, these changes exceeded the thresholds for meaningful improvement (changes exceeding 0.015 for SRMR and 0.01 for CFI and TLI); therefore, we used the ESEM to improve the oblique three-factor model meaningfully. The superiority of the ESEM for the Chinese sample was further supported by the factor loading pattern: items loaded predominantly on their expected factors (with Item 1 of the Stress subscale as the exception), and cross-loadings on non-target factors were small in magnitude (some near zero or negative; see Table 5), indicating good discriminant validity. However, for the Lebanese participants, the ESEM did not yield substantial improvements over the oblique three-factor model. The Lebanese ESEM also showed problematic cross-loading patterns, particularly within the depression subscale, where items failed to load predominantly on their expected factors (see Table 5).

**Table 5:** Standardized Factor Loadings of the DASS-Y from One-Factor CFA and ESEM Models: Lebanese and Chinese Participants.

	One-factor CFA		ESEM					
	Lebanese	Chinese	Lebanese			Chinese		
	Distress		Dep	Anx	Stress	Dep	Anx	Stress
Item 3	0.76	0.82	0.09	0.73	0.10	0.62	0.24	0.01
Item 5	0.79	0.79	−0.01	0.81	−0.06	0.73	−0.05	0.19
Item 10	0.80	0.80	−0.02	0.79	0.06	0.75	0.11	−0.01
Item 13	0.84	0.84	−0.10	0.84	0.04	0.73	0.19	−0.03
Item 16	0.83	0.81	−0.10	0.86	−0.05	0.79	0.01	0.06
Item 17	0.86	0.83	−0.17	0.88	0.01	0.86	0.04	−0.05
Item 21	0.86	0.85	−0.12	0.89	−0.04	0.81	0	0.10
Item 2	0.73	0.73	0.32	0.68	0.06	−0.02	0.86	−0.03
Item 4	0.76	0.76	0.30	0.75	−0.06	0.06	0.84	−0.08
Item 7	0.80	0.75	0.20	0.78	0	−0.07	0.90	0.01
Item 9	0.79	0.86	0.09	0.82	−0.13	0.44	0.45	0.02
Item 15	0.84	0.85	−0.01	0.87	−0.11	0.49	0.35	0.07
Item 19	0.82	0.81	0.04	0.81	0.02	0.14	0.74	0
Item 20	0.80	0.83	0.04	0.83	−0.12	0.34	0.54	0.01
Item 1	0.58	0.74	0	0.47	0.42	−0.01	0.56	0.31
Item 6	0.76	0.77	0.08	0.67	0.33	0.16	0.26	0.45
Item 8	0.75	0.71	0.02	0.66	0.35	0.19	−0.03	0.72
Item 11	0.80	0.73	−0.04	0.76	0.19	0.06	0.20	0.48
Item 12	0.82	0.60	−0.03	0.75	0.26	0.13	0.03	0.58
Item 14	0.79	0.64	0.02	0.76	0.10	−0.02	0.18	0.65
Item 18	0.80	0.76	−0.02	0.75	0.23	0.16	0.07	0.71

Dep = Depression subscale; Anx = Anxiety subscale; Stress = Stress subscale

Following general guidelines [48], and to maintain a consistent analytical strategy across samples, we did not examine the bifactor-ESEM structure. The decision rested on the ESEM results for the Lebanese participants, which showed problematic cross-loading patterns and minimal fit improvements over simpler models. For the bifactor-CFA, the model achieved acceptable fit indices in both countries (see Table 4). The standardized factor loadings reported in S2 Table allowed us to calculate the variance explained by the general factor and the three specific factors (see Table 6). For Lebanese adolescents, the percentage of uncontaminated correlations (PUC) was 0.70, and the explained common variance (ECV) for the general factor was 0.92. These bifactor indices indicate that the variance in the DASS-Y scores was overwhelmingly explained by the general psychological distress factor. The ECVs for the specific factors were 0.04 for depression, 0.09 for anxiety, and 0.14 for stress, suggesting that they contributed little of their own beyond the general factor. Omega hierarchical (ω_H) was 0.95, indicating that the general factor accounted for the great majority of total-score variance.

**Table 6:** Bifactor Indices for the 21-Item and 12-Item DASS-Y in the Lebanese and Chinese Samples.

Sample	Version	PUC	ECV	*ω_*H	ECV_S Dep	ECV_S Anx	ECV_S Str
Lebanese	21-item	0.70	0.92	0.95	0.04	0.09	0.14
Chinese	21-item	0.70	0.83	0.93	0.17	0.09	0.26
Lebanese	12-item	0.73	0.92	0.94	0.06	0.12	0.07
Chinese	12-item	0.73	0.77	0.88	0.05	0.33	0.32

*Note.* PUC = percentage of uncontaminated correlations; ECV = explained common variance; ω_H = omega hierarchical; ECV_S = explained common variance of the specific factor; Dep = depression; Anx = anxiety; Str = stress. Indices were computed from the standardized loadings of the bifactor CFA reported in S2 Table (21-item) and S6 Table (12-item). ECV for the general factor is expressed relative to the total common variance in the model; specific-factor ECVs (ECV_S) are expressed relative to the common variance within the items of that subscale, and therefore do not sum with the general-factor ECV to 1.00.

Within the bifactor-CFA framework for Chinese adolescents, the PUC value was the same (0.70), but the general factor ECV was somewhat lower at 0.83 (ω_H = 0.93). The general factor remained the most dominant. Compared to the Lebanese sample, however, the specific factors made stronger unique contributions in the Chinese sample, with specific-factor ECVs of 0.17 for depression, 0.09 for anxiety, and 0.26 for stress. This greater differentiation was carried by the depression and stress factors; the anxiety factor contributed no more unique variance than in the Lebanese sample. This pattern suggests that depression and stress are somewhat more distinguishable in the Chinese sample than in the Lebanese sample, even though the general factor remains dominant in both contexts.

Based on these findings, we selected different optimal factor structures for the two national contexts. For the Lebanese sample, a one-factor structure was chosen as the most appropriate model. The reasoning was as follows: (a) the one-factor structure achieved acceptable fit; (b) although the oblique three-factor model also showed acceptable fit, its discriminant validity was poor, with interfactor correlations exceeding 0.90; (c) the ESEM did not yield meaningful improvements and was beset with problematic cross-loadings within the subscale; and (d) the bifactor analysis revealed overwhelming general-factor dominance (ECV = 0.92) and negligible specific-factor contributions, suggesting essential unidimensionality in this population. ESEM was selected for the Chinese sample. The reasoning was that (a) the ESEM yielded substantial and meaningful improvements in fit over the simpler models; (b) it produced more differentiated interfactor correlations (from 0.60 to 0.79) than the oblique three-factor CFA, which suffered from poor discriminant validity (correlations between 0.86 and 0.90); and (c) although the bifactor analysis still pointed to general factor dominance, the specific factor contributions were stronger (ECVs = 0.09 to 0.26, with depression and stress accounting for the difference) than in the Lebanese sample, which signaled the presence of meaningful construct-specific variance of the kind that an ESEM structure is well suited to capture.

Reliability was then examined for the conventional scoring structure, in which the three subscales and the total score are used. Although ESEM provided the better representation of the Chinese data, CR and AVE were computed from the simple-structure models in both samples, as described in Section 2.4. CR was satisfactory in both samples, ranging from 0.91 to 0.95 across subscales and reaching 0.97 for the total score in each sample. AVE exceeded 0.50 for every subscale (0.58–0.71) and for the total score (0.63 in the Lebanese and 0.61 in the Chinese sample; S3 Table).

### 3.4 Development of a shortened DASS-Y

Following our evaluation of the 21-item DASS-Y, and given that the one-factor structure was selected for Lebanese participants and the ESEM for Chinese participants, we developed a shortened 12-item version by retaining four items per subscale. The item selection was guided by three criteria, applied simultaneously across both samples: (a) high target factor loadings from both the one-factor CFA and the ESEM solutions (see Table 5 for the factor loadings); (b) minimal cross-loadings on non-target factors in the ESEM solutions; and (c) DIF findings from the Rasch analysis (see Table 3). Items that showed problematic cross-national difficulty contrasts (e.g., Item 5 from the Depression subscale and Item 1 from the Stress subscale), or that showed substantial cross-loadings on non-target factors (e.g., Items 9 and 15 from the Anxiety subscale, which had Depression cross-loadings of 0.44 and 0.49, respectively, in the Chinese ESEM, equaling or exceeding their target Anxiety loadings of 0.45 and 0.35), were excluded. The resulting 12-item instrument retained Items 10, 16, 17, and 21 from the Depression subscale; Items 2, 4, 7, and 19 from the Anxiety subscale; and Items 8, 12, 14, and 18 from the Stress subscale. A note about Item 14 is in order here. Item 14 showed a difficulty contrast exceeding 0.64 logits in the Rasch analysis, but we chose to keep it because it showed acceptable target loadings in both the one-factor CFA (Lebanese = 0.79; Chinese = 0.64) and the Chinese ESEM (Stress = 0.65), with relatively small cross-loadings. The remaining candidate Stress items not already selected (Items 6 and 11) showed less favorable loading patterns. Item 11 had a substantial cross-loading on Anxiety (0.76) that exceeded its target Stress loading (0.19) in the Lebanese ESEM, which signaled weaker factorial clarity across samples. Item 6 showed a relatively lower target Stress loading (0.45) along with notable cross-loadings on Depression (0.16) and Anxiety (0.26) in the Chinese ESEM. The subsequent measurement invariance testing confirmed that the 12-item version achieved acceptable scalar invariance both with and without Item 14 (see Table 6), and we take this to support the robustness of the decision to retain it.

The 12-item version showed psychometric patterns similar to those of the 21-item DASS-Y in terms of model fit across factor structures (see S4 Table in the Supplement File). The unidimensional structure continued to show an inadequate fit for Chinese participants, paralleling what we observed for the 21-item version.

The oblique three-factor structure still fit well in both countries, but it continued to show poor discriminant validity, with latent correlations exceeding 0.90 among Lebanese adolescents. The correlations among the Chinese participants fell within the acceptable range of 0.68 to 0.80, which is an improvement in discriminant validity over the 0.86 to 0.90 range seen in the 21-item version.

The ESEM showed substantial improvement in model fit for the Chinese participants (e.g., ΔRMSEA = 0.018). Beyond the better fit, the factor loadings performed well, with all target loadings exceeding 0.70 and cross-loadings on non-target factors that were small in magnitude, some near zero or negative; this clear simple structure reinforces the discriminant evidence from the latent correlations reported above (see S5 Table). For the Lebanese participants, by contrast, this structure produced only minimal improvements, with negligible changes across fit indices. Although the Depression-subscale factor loadings improved in the 12-item ESEM relative to the 21-item version, certain problems persisted. These included high cross-loadings within the Depression subscale (see S5 Table) and non-significant correlations between anxiety and stress and between depression and stress.

The factor loadings of the bifactor structure are presented in S6 Table. Based on these values, additional bifactor indices were calculated (Table 6). For the Chinese participants, the specific factors of anxiety and stress yielded meaningful ECV (ECV = 0.33 and 0.32, respectively), although the depression factor contributed little (ECV = 0.05). The PUC of 0.73, in combination with the general factor ECV of 0.77 (ω_H = 0.88), suggested that the bifactor model represented a meaningful psychometric structure for Chinese participants. Notably, the anxiety specific factor was substantially stronger here than in the 21-item solution (0.33 vs. 0.09), because the abbreviated form omits the three anxiety items whose specific loadings were near zero or negative. The short form therefore recovers a distinguishable anxiety factor that the full scale obscures.

For Lebanese participants, the picture was different. Specific factors contributed only minimally to the explained variance (ECVs = 0.06 for depression, 0.12 for anxiety, and 0.07 for stress), while the general factor remained dominant (PUC = 0.73, ECV = 0.92, ω_H = 0.94). This pattern matched what was observed in the 21-item version and suggests robust structural consistency across both the full and abbreviated forms of the instrument.

Reliability of the abbreviated version was likewise satisfactory (subscale CR = 0.88–0.92, AVE = 0.65–0.73; total CR = 0.96 and 0.94; total AVE = 0.64 and 0.57, in the Lebanese and Chinese samples respectively; S3 Table), with AVE exceeding the 21-item values in five of six subscale comparisons.

Taken together, these results suggest that the 12-item DASS-Y retains the psychometric integrity of the original instrument while offering a useful tool for efficient screening across national contexts in the future. In short, the unidimensional structure appears to be the most appropriate way to interpret DASS-Y scores in Lebanese adolescents, while both the bifactor and ESEM models provide meaningful interpretations for the Chinese adolescent population.

### 3.5 Measurement invariance

Multi-group invariance testing requires an identical model specification across groups. Neither of the sample-specific solutions identified above could serve this purpose: the one-factor structure preferred for the Lebanese sample fell well short of acceptable fit among Chinese participants (CFI = 0.861, RMSEA = 0.077), and the ESEM solutions were not directly comparable across samples given the divergent loading patterns reported in Table 5. The oblique three-factor CFA was therefore specified in both groups, as the only structure achieving acceptable fit in each sample (see Table 4). The divergence reported in Section 3.3 therefore concerns which model represents each sample most parsimoniously, not whether a common structure holds: the three-factor model was tenable in both samples, and the invariance tests that follow ask whether its parameters are equivalent across them.

The three items showing differential item functioning (Items 1, 5, and 14; Section 3.2) were excluded from the subsequent multi-group CFA, and invariance testing was conducted on the remaining 18 items. The analysis confirmed measurement invariance of this reduced 18-item set across the Lebanese and Chinese adolescent populations (see Table 7). Comparable results were obtained for the 12-item short form: both the full 12-item set and the reduced 11-item set (excluding Item 14) showed acceptable loading and threshold invariance across samples, with ΔCFI, ΔRMSEA, and ΔSRMR values remaining within conventional thresholds [43,44].

**Table 7:** Measurement Invariance Testing of the DASS-Y Across Lebanese and Chinese Participants.

	χ^2^	*Df*	CFI	RMSEA	SRMR	Δχ^2^	Δ*df*	ΔCFI	ΔRMSEA	ΔSRMR
**21-item DASS-Y**										
**Full item set**										
Configural Invariance	1232.02	372	0.944	0.050	0.034	-	-	-	-	-
Loading constrained	1294.72	390	0.941	0.050	0.048	62.70	18	-0.003	0	0.014
Thresholds and loadings constrained	1494.72	408	0.929	0.054	0.054	200.00	18	-0.012	0.004	0.006
**Reduced item set^a^**										
Configural Invariance	872.28	264	0.952	0.050	0.031	-	-	-	-	-
Loading constrained	923.98	279	0.949	0.050	0.043	51.70	15	-0.003	0	0.012
Thresholds and loadings constrained	1051.05	294	0.940	0.053	0.047	127.07	15	-0.009	0.003	0.004
**12-item DASS-Y**										
**Full item set**										
Configural Invariance	142.19	102	0.995	0.021	0.019	-	-	-	-	-
Loading constrained	170.74	111	0.992	0.024	0.033	28.55	9	-0.003	0.003	0.014
Thresholds and loadings constrained	221.57	120	0.987	0.030	0.040	50.83	9	-0.005	0.006	0.007
**Reduced item set^a^**										
Configural Invariance	113.64	82	0.990	0.021	0.017	-	-	-	-	-
Loading constrained	141.70	90	0.992	0.025	0.035	28.06	8	0.002	0.004	0.018
Thresholds and loadings constrained	178.42	98	0.988	0.030	0.039	36.72	8	-0.004	0.005	0.004

*Notes:* CFI = comparative fit index; RMSEA = root mean square error of approximation; SRMR = standardized root mean square residual; ^a^: The reduced item sets exclude items that exhibited differential item functioning in the Rasch-based DIF analysis: Item 5 (Depression subscale) and Items 1 and 14 (Stress subscale) were excluded from the 21-item version, and Item 14 (Stress subscale) was excluded from the 12-item version.

Building on this established invariance, the latent mean comparisons using the 18-item version revealed significant differences in psychological distress levels between samples. The Lebanese adolescents showed significantly higher latent factor scores than their Chinese counterparts across all three dimensions: depression (*t* = 14.34, *p* < .001), anxiety (*t* = 15.54, *p* < .001), and stress (*t* = 12.27, *p* < .001). For the Lebanese sample in particular, where the specific factors accounted for little variance beyond the general distress factor, these comparisons are best read as reflecting a common elevation in general distress rather than three independent dimensional differences. Considered together, these results indicate substantially higher levels of psychological distress among Lebanese adolescents than among Chinese adolescents.

Two further sets of supplementary analyses examined invariance within samples. Because socioeconomic status was not measured directly, the first tested invariance across the two Chinese provincial subsamples (S7 Table); Guangdong and Jiangxi differ substantially in level of economic development, so this provides an indirect check on whether the measurement model is sensitive to regional economic context. The second tested invariance across male and female adolescents in each country (S8 Table). In every comparison, both the 21-item and the 12-item versions showed full scalar invariance, with all changes in fit indices remaining within conventional thresholds (|ΔCFI| ≤ 0.002, |ΔRMSEA| ≤ 0.002, |ΔSRMR| ≤ 0.006). Item loadings and intercepts therefore did not vary with regional economic context, although regional development is a coarse proxy for individual socioeconomic circumstances, and the results support the validity of cross-national comparisons despite the unequal sex composition of the two samples.

## 4. Discussion

The cross-national validity of psychological assessment instruments remains a central concern in adolescent mental health research, and the need for comparable measurement tools has grown as international collaboration in mental health science has intensified. Although the DASS-Y has been widely adopted to capture depression, anxiety, and stress as a continuum of negative affect, its measurement invariance between Middle Eastern and East Asian adolescents has not, to our knowledge, been tested. Moreover, most prior validations have relied on a single analytic approach, leaving more complex factor structures unexamined and brief screening forms undeveloped. This study addresses these gaps. The psychometric properties of the DASS-Y in Lebanese and Chinese adolescent samples were evaluated through Rasch analysis, competing factor models (CFA, ESEM, bifactor, and bifactor-ESEM), and DIF analysis. An abbreviated 12-item version was developed and validated for screening purposes. Because the two samples differed in sex composition, supplementary analyses were conducted to test measurement invariance across boys and girls within each country to guard against interpretive bias. The optimal factor structure differed between contexts: a unidimensional model best fit the Lebanese data, whereas an ESEM solution better represented the multidimensional structure of distress in the Chinese sample. Three items exhibited substantial DIF and compromised direct cross-national comparisons. The 12-item short form showed psychometric properties broadly comparable to those of the full 21-item version.

### 4.1 Cross-national differences in psychological distress: The role of sociopolitical context

Before interpreting the cross-national differences observed in the present study, an important framing point should be made explicit. The Lebanese and Chinese samples differ not only in national and linguistic context but also in their acute environmental stress exposure at the time of data collection. These two factors cannot be disentangled given the present design. The findings reported here, then, should be read as a description of how the DASS-Y functions, and of how distress is reported, across two real-world national contexts at a specific historical moment. They are not a clean test of culture-specific or stress-specific effects. This framing applies as much to the differences in distress levels discussed in this section as to the differences in factor structure and item functioning discussed below.

Using the cut-off criteria of the DASS-Y [10], the Lebanese adolescents in our sample showed visibly higher rates of psychological distress across all domains compared to their Chinese counterparts: depression (52.2% vs. 27.0%), anxiety (55.9% vs. 24.9%), and stress (26.5% vs. 6.8%). These rates likely reflect, at least in part, Lebanon’s sociopolitical context and the strains that come with it. The Lebanese data were collected in November 2023, a period of heightened tensions between Lebanon and Israel, and it is reasonable to suppose that this contributed to the elevated distress levels we observed. Merrilees et al. [49] have documented how exposure to political violence and instability can produce substantial psychological distress in adolescents. The ongoing economic and social challenges in Lebanon almost certainly serve as additional stressors that affect adolescent mental health [29]. These contextual factors help to make sense of the substantial differences in distress levels between the Lebanese and Chinese samples in the present study. Chinese adolescents, in contrast, face a different set of pressures, which center largely on academic achievement and familial expectations [30], although they live within a more politically stable environment that offers a degree of economic security. With all this in mind, and consistent with the framing noted above, the magnitude of the cross-national difference in distress levels reported here cannot be uniquely attributed to either national context or to differential stress exposure. A within-population longitudinal comparison of the same Lebanese adolescents across periods of relative stability and acute sociopolitical strain, such as before and after a major regional escalation, would help to isolate the contribution of acute stress exposure from national-context effects.

### 4.2 Rasch analysis findings

The application of Rasch analysis to the DASS-Y adds useful evidence for its psychometric evaluation. Although Rasch analysis has been applied in Chinese [11] and Indonesian [15] samples, it has not previously been used to compare item functioning across national samples. Our findings for the Chinese participants align with those of Cao et al. [11], who likewise reported acceptable item validity among Chinese primary and middle school students.

Three items showed differential item functioning across the samples, and the pattern was content-specific rather than subscale-wide. Item 14 (“I got annoyed when people interrupted me”) produced the largest contrast at 1.02 logits and was endorsed far more readily by Chinese adolescents than their overall distress level would predict. More generally, non-equivalence within the stress subscale was concentrated in items describing irritability and over-reaction, while items describing tension and difficulty relaxing transferred well across samples. The direction of these differences, however, was not systematic: Items 14 and 18 were easier to endorse for Chinese adolescents, whereas Items 1, 6 and 11 were easier for Lebanese adolescents. Irritability content is therefore where the two populations diverge most in degree, without a uniform shift in either direction.

The poor performance of Item 1 (“I got upset about little things”) on the Stress subscale among the Lebanese adolescents is, in our view, especially worth dwelling on, because it may reveal something important about how psychological distress is experienced and interpreted in this context. There are two complementary readings of this finding. The first concerns the construct validity of the item. Jovanović [19] has observed that this item often loads on anxiety factors in psychometric analyses and that it is even used to measure anxiety in other instruments. There appears, then, to be a conceptual overlap baked into the item itself. The cross-loading pattern we observed among the Lebanese adolescents is therefore not unexpected, and the elevated outfit statistics in the Rasch analysis lend further support to the conclusion that this item is measuring something distinct from the other stress items in this population. The second reading is contextual, and to our minds it is the more important of the two. The finding likely reflects the way chronic sociopolitical instability shapes cognitive appraisal processes. In a context of prolonged conflict and uncertainty, of the kind Lebanese adolescents have been navigating, the concept of “little things” may lose its meaning or come to be reframed in ways that complicate the response. When one is living under constant threat, the threshold for what counts as a “minor” versus a “significant” stressor shifts dramatically. For these adolescents, everyday irritants may simply fail to register as separable from pervasive background stress; alternatively, those same irritants may trigger disproportionate reactions because of cumulative strain.

The fact that this item functioned well among Chinese participants but not among Lebanese adolescents is informative. This difference is unlikely to be attributable to collectivism, which is shared by both populations. A more plausible interpretation is that some combination of the specific sociopolitical context of Lebanese youth, language-level differences in how the everyday phrase “little things” is interpreted semantically and pragmatically in Arabic versus Chinese, and the longer-term developmental influences of chronic environmental stress on emotion vocabulary all played a role. This interpretation aligns with trauma-informed perspectives on assessment, which recognize how chronic exposure to adversity can fundamentally alter cognitive appraisal processes [49]. It also resonates with sociolinguistic perspectives that emphasize how emotion-related vocabulary is acquired and refined within specific linguistic and developmental contexts [50].

### 4.3 Factor structure variations across national contexts

Factor structure analyses revealed notable differences between the two national contexts, serving as a reminder that sociopolitical and contextual factors matter when evaluating measurement models.

For Lebanese adolescents, a unidimensional model showed an acceptable fit, and a bifactor model with a dominant general factor of psychological distress best represented the data structure, with little variance attributable to the specific factors. The oblique three-factor structure also showed an acceptable fit in the Lebanese sample, consistent with the findings of Obeid et al. [17] for Lebanese adolescents and Shabani et al. [16] for Iranian children and adolescents. However, the extremely high interfactor correlations observed in the present study (exceeding.90) point to poor discriminant validity, and this conclusion is reinforced by the bifactor indices (PUC and ECV) reported earlier. Overall, a unidimensional structure represents the most parsimonious model for the Lebanese context.

For Chinese adolescents, both ESEM and bifactor models (particularly for the 12-item version) provided meaningful interpretations, with the specific factors making more substantial contributions to the explained variance than in the Lebanese sample, where they were negligible. Which factors contributed differed by version: depression and stress in the 21-item solution, anxiety and stress in the 12-item solution. The bifactor-ESEM model was not evaluated in either version, for different reasons. For the 21-item version it was not estimated: the specific factors in the Lebanese bifactor solution were negligible, indicating essential unidimensionality, so freely estimating cross-loadings in addition to a general factor was not warranted. For the 12-item version it was specified but failed to converge in the Chinese sample. Even with these limitations, the findings partly align with those of Jovanović [19], who found that a bifactor-ESEM model best represented the DASS-Y structure in Serbian adolescents and established measurement invariance with an Australian sample. The alignment is partial because, in the present study, the ESEM and bifactor approaches each performed well in isolation, whereas their combination (bifactor-ESEM) did not prove viable in the Chinese sample. The ESEM and bifactor-ESEM approaches remain relatively underutilized in DASS-Y validation, and this gap warrants attention in future psychometric work. As Van Zyl and Ten Klooster [47] noted, ESEM often provides more accurate representations of the factor structure for psychological constructs by accounting for the small but meaningful cross-loadings that exist between theoretically related but distinct factors. This flexibility is desirable for an instrument such as the DASS-Y, where depression, anxiety, and stress share conceptual territories.

A further observation concerns the anxiety factor. In the 21-item solution, the Chinese anxiety specific factor accounted for almost no variance beyond the general factor (ECV = 0.09), a value indistinguishable from the Lebanese estimate. In the 12-item solution it accounted for the most (0.33). The difference is attributable to item selection: the three anxiety items omitted from the short form were precisely those whose specific loadings were near zero or negative. Their removal leaves a set of anxiety items that share reliable variance beyond general distress. The abbreviated form is therefore not simply a shorter version of the full scale; for this sample it yields a more differentiated measurement structure, which is an unusual property for a short form and one that warrants replication before it is relied upon.

### 4.4 Implications for cut-off scores

The DASS-Y is used in practice mainly to flag adolescents whose distress warrants attention, and that use rests on the cut-off scores proposed by Szabo and Lovibond [10]. The present results speak to how those thresholds behave in the two contexts rather than to their revision; no diagnostic criterion measure was administered, so sensitivity and specificity could not be estimated.

Two aspects of score interpretation should be kept apart. The elevated distress observed among Lebanese adolescents is substantive rather than an artifact of item functioning: with the three non-equivalent items set aside, the remaining 18 were invariant across samples and latent means were significantly higher in the Lebanese sample (Section 3.5). The raw-score metric on which the cut-offs operate is another matter, since those items retain their weight in the conventionally computed total. The classification rates in Section 3.1 (46.0% versus 12.7% above the total-score threshold of 23) therefore track the direction of the difference dependably without being calibrated to a common scale. Subscale thresholds are more problematic still, presupposing three separable dimensions that the Lebanese data do not support.

The published cut-offs thus remain serviceable within a country while carrying little warrant for between-country comparison of prevalence, and the total score is the sounder basis for classification wherever the structure proves essentially unidimensional. Country-specific thresholds for both forms, derived against a diagnostic criterion, are a task for subsequent work.

### 4.5 Limitations

Several limitations should be noted. The most fundamental is that national context, acute environmental stress exposure, and language are confounded in the present design. The Lebanese sample was collected during a period of heightened regional tensions, the Chinese sample under conditions of relative political stability, and the two groups responded to different translations. Differences in distress levels, factor structure, and item functioning therefore cannot be attributed uniquely to any one of these. Language may operate through two routes the design cannot separate: Arabic and Chinese demarcate the distress lexicon differently, and chronic adversity may itself shape the development of emotional vocabulary, so the unidimensional structure among Lebanese adolescents may reflect longer-term influences on emotion language as well as distress at the time of assessment. Accordingly, the findings have been framed in terms of national and sociopolitical context rather than culture. Cognitive interviews into how adolescents interpret specific items in their native languages, and within-population longitudinal comparison across periods of relative stability and acute strain, would help to separate these influences.

A second set of limitations concerns sampling. Sociodemographic variables such as household income and parental education were not collected in either protocol, so socioeconomic differences could not be modelled; the sampling frames indicate the range represented rather than measuring it, with the Chinese sample drawn entirely from urban, public, academic-track schools and Lebanese online recruitment requiring device ownership and connectivity. Invariance across the two Chinese provinces, which differ substantially in economic development, suggests the measurement model was not sensitive to regional economic context, but no equivalent check was possible in Lebanon. Recruitment also differed in kind: online snowball sampling in Lebanon against school-based administration in China, where intact classes were selected by administrators rather than at random. Neither design permits a participation rate to be computed, and the clustering of Chinese responses within classes and schools was not modelled, as the analyses concern measurement structure rather than population estimates. The samples further differed in composition, the Lebanese being more heterogeneous in age and comprising 63.2% girls against 47.5%; to the extent that distress levels differ by sex, this imbalance may contribute to the observed difference in mean levels, although the items functioned equivalently across sex within each sample.

Third, the scope of measurement was limited. Both samples were non-clinical, all measures were self-reported, and no diagnostic criterion was administered, so the sensitivity and specificity of the published cut-offs could not be evaluated and no country-specific thresholds could be derived (see Section 4.4).

Finally, the 12-item form was derived and evaluated in the same two samples without independent cross-validation. Its properties, including the more differentiated anxiety factor observed in the Chinese sample, may partly reflect sample-specific variance, and replication is needed before the form is adopted for routine screening.

## 5. Conclusion

This study took up the three gaps identified at the outset: the DASS-Y had never been tested for measurement equivalence between Middle Eastern and East Asian adolescents, prior validations had primarily relied on single analytic approaches, and no brief form had been evaluated across national contexts. Examining Lebanese and Chinese samples through Rasch analysis, competing factor models, and DIF testing showed that equivalence cannot be taken for granted even between populations sharing broad cultural features such as collectivism, since Lebanese adolescents responded as though a single distress construct was being tapped whereas Chinese adolescents differentiated depression, anxiety, and stress. The scale itself performed strongly in both settings, with CR ranging from 0.88 to 0.97 across subscales and total scores at both lengths, AVE between 0.57 and 0.73, and Rasch infit and outfit values within the accepted range for all items but one, so this structural divergence is substantive rather than a product of weak measurement or item quality, though whether it lies in adolescents’ experience of distress or in the language-specific rendering of the items cannot be fully separated here. The multi-method design proved its worth here, as no single approach would have revealed the full pattern: three items, concentrated in the stress subscale and including the single misfitting item, functioned inconsistently across samples, and although their removal restored invariance and latent means confirmed that the higher distress among Lebanese adolescents is substantive, the raw-score metric beneath the published cut-offs remains uncalibrated across countries, leaving those thresholds better read as within-country reference points. The newly developed 12-item form, finally, retained the reliability and psychometric integrity of the full scale and even yielded a more clearly differentiated anxiety factor in the Chinese sample, offering a strong candidate for the brief cross-national screening tool the literature lacks. Replication in further contexts, with criterion-referenced work to establish country-specific thresholds, is the natural next step.

## Supporting information

S1 Table Published Psychometric Evaluations of the DASS-Y. (DOCX)

S2 Table Standardized Factor Loadings Derived from the Bifactor Model for the 21-item DASS-Y. (DOCX)

S3 Table Reliability of the 21-Item and 12-Item DASS-Y in the Lebanese and Chinese Samples. (DOCX)

S4 Table Model Fit Indices for Competing Factor Structures of the 12-Item DASS-Y Across Lebanese and Chinese Participants. (DOCX)

S5 Table Standardized Factor Loadings of the 12-item DASS-Y: from One-Factor CFA and ESEM Models: Lebanese and Chinese Participants. (DOCX)

S6 Table Standardized Factor Loadings Derived from the Bifactor Model for the 12-item DASS-Y. (DOCX)

S7 Table Measurement Invariance Tests for the 21-Item and 12-Item Scales Across Guangdong and Jiangxi Provinces in China. (DOCX)

S8 Table Measurement Invariance Testing of the DASS-Y Across Male and Female Adolescents Within Each National Sample. (DOCX)

S1 Checklist Inclusivity in global research. (DOCX)
