# Supplementary material for: Cross-national validation of the Depression, Anxiety and Stress Scale for Youth (DASS-Y) in Lebanese and Chinese adolescents
Source: PLoS One. 2026 Oct 21;21(10):e0360807. doi: 10.1371/journal.pone.0360807 (PMC13686488; doi:10.1371/journal.pone.0360807)
Supplement: Supplementary file 1 [file pone.0360807.s001.docx]

S1 Table. Published Psychometric Evaluations of the DASS-Y

| Study (country; language) | Sample | Analytic approach | Measurement invariance tested | Abbreviated form |
| --- | --- | --- | --- | --- |
| Szabó & Lovibond, 2022 (Australia; English) | *N* = 2,121, aged 7–18, 61% female | CFA (ML); multiple regression | None | No |
| Cao et al., 2023 (China; Chinese) | *N =* 1,507 primary and 1,131 middle-school students (age and sex distribution not reported) | Rasch; CFA | None | No |
| Jiang et al., 2024 (China; Chinese) | *N* = 326, aged 14–18, *M* = 15.72 (*SD* = 0.82), 50% female, one urban and one rural secondary school | CFA (ML) | None | No |
| Sarfika et al., 2024 (Indonesia; Bahasa Indonesia) | *N* = 255, 79.6% female, grades 10–12, two public senior high schools | Content validity; EFA (PCA, varimax); CFA (ML); two-week test–retest ICC | None | No |
| Śliwerski et al., 2025 (Poland; Polish) | *N* = 344, aged 9–17, *M* = 13.84 (*SD* = 2.36), 55.2% female, four schools (two primary, one combined, one secondary) | CFA (MLR) | Across sex: metric supported; scalar not fully supported | No |
| Shabani et al., 2025 (Iran; Persian) | *N* = 1,277, aged 7–18, *M* = 13.22 (*SD* = 3.07), 55.1% female, 15 primary schools and secondary schools | CFA (ML) | Across sex and age band: scalar supported | No |
| Jovanović, 2025 (Serbia and Australia; Serbian, English) | *N* = 421, aged 15–19, *M* = 16.86 (*SD* = 0.89), 48% female, three public high schools | CFA (WLSMV) | Across countries (Serbia–Australia) and sex: scalar supported | No |
| Obeid et al., 2025 (Lebanon; Arabic) | *N* = 758, aged 12-18, *M* = 15.95 (*SD* = 1.78), 63.2% female, snowball recruitment | CFA(ML) | Across sex: scalar supported | Yes |
| Kurnianto et al., 2025 (Indonesia; Bahasa Indonesia) | *N =* 355, aged 10–18, 63.1% female, eight schools (four primary, four secondary) | Rasch | DIF across sex, age band, educational level and school type | No |
| Wang et al., 2026 (China; Chinese) | Two studies. Study 1: *N* = 736, Grade 11 students, 48% female, two public senior high schools; Study 2: *N* = 1,676, Grades 7–9, 46.96% female, urban public schools | CFA (WLSMV); Rasch | Across time (3-month): scalar supported. Across educational level (primary vs. middle school): partial scalar | No |

*Notes.* EFA = exploratory factor analysis; PCA = principal component analysis; CFA = confirmatory factor analysis; ESEM = exploratory structural equation modeling; ML = maximum likelihood; MLR = maximum likelihood with robust (Huber–White) standard errors; WLSMV = weighted least squares mean- and variance-adjusted; ICC = intraclass correlation coefficient; DIF = differential item functioning
