# Supplementary material for: Cross-national validation of the Depression, Anxiety and Stress Scale for Youth (DASS-Y) in Lebanese and Chinese adolescents
Source: PLoS One. 2026 Oct 21;21(10):e0360807. doi: 10.1371/journal.pone.0360807 (PMC13686488; doi:10.1371/journal.pone.0360807)
Supplement: Supplementary file 2 [file pone.0360807.s002.docx]

S2 Table. Standardized Factor Loadings Derived from the Bifactor Model for the 21-item DASS-Y

|  | **Lebanese** | | | | **Chinese** | | | |
| --- | --- | --- | --- | --- | --- | --- | --- | --- |
|  | **G factor** | **Dep** | **Anx** | **Stress** | **G factor** | **Dep** | **Anx** | **Stress** |
| **Depression subscale** |  |  |  |  |  |  |  |  |
| Item 3 | 0.77 | 0.39 |  |  | 0.80 | 0.20 |  |  |
| Item 5 | 0.80 | 0.11 |  |  | 0.74 | 0.36 |  |  |
| Item 10 | 0.81 | 0.01 |  |  | 0.77 | 0.29 |  |  |
| Item 13 | 0.85 | -0.03 |  |  | 0.81 | 0.27 |  |  |
| Item 16 | 0.84 | -0.06 |  |  | 0.75 | 0.41 |  |  |
| Item 17 | 0.87 | -0.09 |  |  | 0.77 | 0.40 |  |  |
| Item 21 | 0.87 | -0.14 |  |  | 0.78 | 0.44 |  |  |
| **Anxiety subscale** |  |  |  |  |  |  |  |  |
| Item 2 | 0.70 |  | 0.31 |  | 0.75 |  | 0.39 |  |
| Item 4 | 0.74 |  | 0.40 |  | 0.78 |  | 0.35 |  |
| Item 7 | 0.78 |  | 0.23 |  | 0.78 |  | 0.32 |  |
| Item 9 | 0.78 |  | 0.18 |  | 0.89 |  | -0.12 |  |
| Item 15 | 0.83 |  | 0.12 |  | 0.89 |  | -0.20 |  |
| Item 19 | 0.81 |  | 0.11 |  | 0.82 |  | 0.24 |  |
| Item 20 | 0.80 |  | 0.17 |  | 0.86 |  | 0.01 |  |
| **Stress subscale** |  |  |  |  |  |  |  |  |
| Item 1 | 0.56 |  |  | 0.37 | 0.73 |  |  | 0.17 |
| Item 6 | 0.73 |  |  | 0.37 | 0.75 |  |  | 0.15 |
| Item 8 | 0.72 |  |  | 0.34 | 0.65 |  |  | 0.56 |
| Item 11 | 0.79 |  |  | 0.21 | 0.72 |  |  | 0.16 |
| Item 12 | 0.80 |  |  | 0.27 | 0.55 |  |  | 0.44 |
| Item 14 | 0.77 |  |  | 0.13 | 0.59 |  |  | 0.48 |
| Item 18 | 0.78 |  |  | 0.27 | 0.70 |  |  | 0.52 |

*Notes.* G factor = general factor in the bifactor model; Dep = Depression subscale; Anx = Anxiety subscale; Stress = Stress subscale
