# Supplementary material for: Cross-national validation of the Depression, Anxiety and Stress Scale for Youth (DASS-Y) in Lebanese and Chinese adolescents
Source: PLoS One. 2026 Oct 21;21(10):e0360807. doi: 10.1371/journal.pone.0360807 (PMC13686488; doi:10.1371/journal.pone.0360807)
Supplement: Supplementary file 3 [file pone.0360807.s003.docx]

S3 Table. Reliability of the 21-Item and 12-Item DASS-Y in the Lebanese and Chinese Samples

|  | **Lebanese** | | **Chinese** | |
| --- | --- | --- | --- | --- |
|  | CR | AVE | CR | AVE |
| **21-item version** |  |  |  |  |
| Depression | 0.94 | 0.69 | 0.95 | 0.71 |
| Anxiety | 0.93 | 0.65 | 0.94 | 0.69 |
| Stress | 0.92 | 0.62 | 0.91 | 0.58 |
| Total scale | 0.97 | 0.63 | 0.97 | 0.61 |
| **12-item version** |  |  |  |  |
| Depression | 0.92 | 0.73 | 0.92 | 0.73 |
| Anxiety | 0.88 | 0.65 | 0.91 | 0.72 |
| Stress | 0.88 | 0.65 | 0.88 | 0.65 |
| Total scale | 0.96 | 0.64 | 0.94 | 0.57 |

*Notes.* CR = composite reliability; AVE = average variance extracted. CR is equivalent to McDonald's ω for congeneric item sets and is reported here under the single label. Subscale values were computed from the standardized loadings of the oblique three-factor model; total-scale values were computed from the one-factor model. Values of CR > 0.70 and AVE > 0.50 are conventionally regarded as satisfactory.
