# Supplementary material for: Cross-national validation of the Depression, Anxiety and Stress Scale for Youth (DASS-Y) in Lebanese and Chinese adolescents
Source: PLoS One. 2026 Oct 21;21(10):e0360807. doi: 10.1371/journal.pone.0360807 (PMC13686488; doi:10.1371/journal.pone.0360807)
Supplement: Supplementary file 4 [file pone.0360807.s004.docx]

S4 Table. Model Fit Indices for Competing Factor Structures of the 12-Item DASS-Y Across Lebanese and Chinese Participants

|  | ***χ^2^* (*df*)** | **CFI** | **TLI** | **RMSEA; 90% CI** | **SRMR** |
| --- | --- | --- | --- | --- | --- |
| **Lebanese** |  |  |  |  |  |
| One-factor | 144.49 (54) | 0.974 | 0.968 | 0.047; 0.041-0.053 | 0.025 |
| Three-factor | 73.44 (51) | 0.994 | 0.992 | 0.024; 0.015-0.032 | 0.017 |
| Bifactor model | 55.05 (42) | 0.996 | 0.994 | 0.020; 0.006-0.030 | 0.012 |
| ESEM | 46.22 (33) | 0.996 | 0.992 | 0.023; 0.010-0.034 | 0.011 |
| Bifactor ESEM | 33.51 (24) | 0.997 | 0.992 | 0.023; 0.002-0.036 | 0.009 |
| **Chinese** |  |  |  |  |  |
| One-factor | 718.91 (54) | 0.839 | 0.804 | 0.108; 0.103-0.112 | 0.068 |
| Three-factor | 68.75 (51) | 0.996 | 0.994 | 0.018; 0.009-0.025 | 0.020 |
| Bifactor model | 51.49 (42) | 0.998 | 0.996 | 0.015; 0.000-0.023 | 0.015 |
| ESEM | 27.94 (33) | 0.999 | 0.999 | 0.000; 0.000-0.011 | 0.007 |
| Bifactor ESEM | Not converged |  |  |  |  |

*Notes.* CFI = comparative fit index; TLI = Tucker-Lewis Index; RMSEA = root mean square error of approximation; SRMR = standardized root mean square residual; CI = confidence interval; ESEM = Exploratory Structural Equation Modeling
