# Supplementary material for: Cross-national validation of the Depression, Anxiety and Stress Scale for Youth (DASS-Y) in Lebanese and Chinese adolescents
Source: PLoS One. 2026 Oct 21;21(10):e0360807. doi: 10.1371/journal.pone.0360807 (PMC13686488; doi:10.1371/journal.pone.0360807)
Supplement: Supplementary file 5 [file pone.0360807.s005.docx]

S5 Table. Standardized Factor Loadings of the 12-item DASS-Y: from One-Factor CFA and ESEM Models: Lebanese and Chinese Participants

|  | **One-factor CFA** | | **ESEM** | | | | | |
| --- | --- | --- | --- | --- | --- | --- | --- | --- |
|  | **Lebanese** | **Chinese** | **Lebanese** | | | **Chinese** | | |
|  | **Distress** | | **Dep** | **Anx** | **Stress** | **Dep** | **Anx** | **Stress** |
| Item 10 | 0.80 | 0.80 | 0.70 | 0.79 | 0.06 | 0.75 | 0.11 | -0.01 |
| Item 16 | 0.83 | 0.81 | 0.84 | 0.86 | -0.05 | 0.79 | 0.01 | 0.06 |
| Item 17 | 0.86 | 0.83 | 0.95 | 0.88 | 0.01 | 0.86 | 0.04 | -0.05 |
| Item 21 | 0.86 | 0.85 | 0.90 | 0.89 | -0.04 | 0.81 | 0 | 0.10 |
| Item 2 | 0.73 | 0.73 | -0.03 | 0.68 | 0.06 | -0.02 | 0.86 | -0.03 |
| Item 4 | 0.76 | 0.76 | 0.03 | 0.75 | -0.06 | 0.06 | 0.84 | -0.08 |
| Item 7 | 0.80 | 0.75 | 0.17 | 0.78 | 0 | -0.07 | 0.90 | 0.01 |
| Item 19 | 0.82 | 0.81 | 0.49 | 0.81 | 0.02 | 0.14 | 0.74 | 0 |
| Item 8 | 0.75 | 0.71 | 0.02 | 0.66 | 0.35 | 0.19 | -0.03 | 0.72 |
| Item 12 | 0.82 | 0.60 | -0.03 | 0.75 | 0.26 | 0.13 | 0.03 | 0.58 |
| Item 14 | 0.79 | 0.64 | 0.02 | 0.76 | 0.10 | -0.02 | 0.18 | 0.65 |
| Item 18 | 0.80 | 0.76 | -0.02 | 0.75 | 0.23 | 0.16 | 0.07 | 0.71 |

*Notes.* Dep = Depression subscale; Anx = Anxiety subscale; Stress = Stress subscale; ESEM = Exploratory Structural Equation Modeling
