# Supplementary material for: Cross-national validation of the Depression, Anxiety and Stress Scale for Youth (DASS-Y) in Lebanese and Chinese adolescents
Source: PLoS One. 2026 Oct 21;21(10):e0360807. doi: 10.1371/journal.pone.0360807 (PMC13686488; doi:10.1371/journal.pone.0360807)
Supplement: Supplementary file 6 [file pone.0360807.s006.docx]

S6 Table. Standardized Factor Loadings Derived from the Bifactor Model for the 12-item DASS-Y

|  | **Lebanese** | | | | **Chinese** | | | |
| --- | --- | --- | --- | --- | --- | --- | --- | --- |
|  | **G factor** | **Dep** | **Anx** | **Stress** | **G factor** | **Dep** | **Anx** | **Stress** |
| **Depression subscale** |  |  |  |  |  |  |  |  |
| Item 10 | 0.81 | 0.09 |  |  | 0.81 | 0.06 |  |  |
| Item 16 | 0.82 | 0.21 |  |  | 0.83 | 0.22 |  |  |
| Item 17 | 0.84 | 0.25 |  |  | 0.83 | 0.26 |  |  |
| Item 21 | 0.85 | 0.27 |  |  | 0.89 | 0.15 |  |  |
| **Anxiety subscale** |  |  |  |  |  |  |  |  |
| Item 2 | 0.71 |  | 0.37 |  | 0.67 |  | 0.50 |  |
| Item 4 | 0.75 |  | 0.32 |  | 0.70 |  | 0.51 |  |
| Item 7 | 0.78 |  | 0.25 |  | 0.67 |  | 0.51 |  |
| Item 19 | 0.82 |  | 0.09 |  | 0.75 |  | 0.41 |  |
| **Stress subscale** |  |  |  |  |  |  |  |  |
| Item 8 | 0.74 |  |  | 0.24 | 0.70 |  |  | 0.51 |
| Item 12 | 0.81 |  |  | 0.30 | 0.58 |  |  | 0.40 |
| Item 14 | 0.80 |  |  | 0.03 | 0.63 |  |  | 0.42 |
| Item 18 | 0.80 |  |  | 0.19 | 0.73 |  |  | 0.47 |

*Notes.* G factor = general factor in the bifactor model; Dep = Depression subscale; Anx = Anxiety subscale; Stress = Stress subscale
