# Supplementary material for: Cross-national validation of the Depression, Anxiety and Stress Scale for Youth (DASS-Y) in Lebanese and Chinese adolescents
Source: PLoS One. 2026 Oct 21;21(10):e0360807. doi: 10.1371/journal.pone.0360807 (PMC13686488; doi:10.1371/journal.pone.0360807)
Supplement: Supplementary file 7 [file pone.0360807.s007.docx]

S7 Table. Measurement Invariance Tests for the 21-Item and 12-Item Scales Across Guangdong and Jiangxi Provinces in China

|  | **χ^2^** | ***df*** | **CFI** | **RMSEA** | **SRMR** | **Δχ^2^** | **Δ*df*** | **ΔCFI** | **ΔRMSEA** | **ΔSRMR** |
| --- | --- | --- | --- | --- | --- | --- | --- | --- | --- | --- |
| **21 items** |  |  |  |  |  |  |  |  |  |  |
| Configural Invariance | 1297.033 | 372 | .917 | 0.069 | .042 | - | - | — | — | — |
| Loading constrained | 1341.932 | 390 | .916 | 0.068 | .045 | 44.899 | 18 | −.001 | −.002 | .003 |
| Thresholds and loadings constrained | 1391.983 | 408 | .915 | 0.067 | .045 | 50.051 | 18 | −.001 | −.001 | .000 |
| **12 items** |  |  |  |  |  |  |  |  |  |  |
| Configural Invariance | 139.270 | 102 | .993 | 0.035 | .023 | - | - | — | — | — |
| Loading constrained | 150.268 | 111 | .993 | 0.034 | .025 | 10.998 | 9 | .000 | −.001 | .002 |
| Thresholds and loadings constrained | 166.163 | 120 | .992 | 0.035 | .025 | 15.895 | 9 | −.001 | .001 | .000 |

*Notes:* CFI = comparative fit index; RMSEA = root mean square error of approximation; SRMR = standardized root mean square residual
