# Supplementary material for: Cross-national validation of the Depression, Anxiety and Stress Scale for Youth (DASS-Y) in Lebanese and Chinese adolescents
Source: PLoS One. 2026 Oct 21;21(10):e0360807. doi: 10.1371/journal.pone.0360807 (PMC13686488; doi:10.1371/journal.pone.0360807)
Supplement: Supplementary file 8 [file pone.0360807.s008.docx]

S8 Table. Measurement Invariance Testing of the DASS-Y Across Male and Female Adolescents Within Each National Sample

|  | **χ^2^** | ***df*** | **CFI** | **RMSEA** | **SRMR** | **Δχ^2^** | **Δ*df*** | **ΔCFI** | **ΔRMSEA** | **ΔSRMR** |
| --- | --- | --- | --- | --- | --- | --- | --- | --- | --- | --- |
| **Lebanese** |  |  |  |  |  |  |  |  |  |  |
| **21 items** |  |  |  |  |  |  |  |  |  |  |
| Configural Invariance | 575.55 | 372 | 0.971 | 0.038 | 0.031 | - | - | - | - | - |
| Loading constrained | 599.42 | 390 | 0.970 | 0.038 | 0.035 | 23.87 | 18 | -0.001 | 0 | 0.004 |
| Thresholds and loadings constrained | 622.35 | 408 | 0.969 | 0.037 | 0.036 | 22.93 | 18 | -0.001 | -0.001 | 0.001 |
| **12 items** |  |  |  |  |  |  |  |  |  |  |
| Configural Invariance | 134.09 | 102 | 0.991 | 0.029 | 0.023 |  |  |  |  |  |
| Loading constrained | 144.79 | 111 | 0.991 | 0.028 | 0.027 | 10.7 | 9 | 0 | -0.001 | 0.004 |
| Thresholds and loadings constrained | 155.09 | 120 | 0.990 | 0.028 | 0.028 | 10.3 | 9 | -0.001 | 0 | 0.001 |
| **Chinese** |  |  |  |  |  |  |  |  |  |  |
| **21 items** |  |  |  |  |  |  |  |  |  |  |
| Configural Invariance | 1127.79 | 372 | 0.915 | 0.062 | 0.044 | - | - | - | - | - |
| Loading constrained | 1153.06 | 390 | 0.914 | 0.061 | 0.049 | 25.27 | 18 | -0.001 | -0.001 | 0.005 |
| Thresholds and loadings constrained | 1191.11 | 408 | 0.912 | 0.060 | 0.049 | 38.05 | 18 | -0.002 | -0.001 | 0 |
| **12 items** |  |  |  |  |  |  |  |  |  |  |
| Configural Invariance | 133.24 | 102 | 0.994 | 0.024 | 0.025 |  |  |  |  |  |
| Loading constrained | 142.81 | 111 | 0.994 | 0.023 | 0.031 | 9.57 | 9 | 0 | -0.001 | 0.006 |
| Thresholds and loadings constrained | 155.19 | 120 | 0.993 | 0.023 | 0.032 | 12.38 | 9 | -0.001 | 0 | 0.001 |

*Notes:* CFI = comparative fit index; RMSEA = root mean square error of approximation; SRMR = standardized root mean square residual
